# Impact of fibre and red/processed meat intake on treatment outcomes among patients with chronic inflammatory diseases initiating biological therapy: A prospective cohort study

**DOI:** 10.3389/fnut.2022.985732

**Published:** 2022-10-13

**Authors:** Silja H. Overgaard, Signe B. Sørensen, Heidi L. Munk, Anders B. Nexøe, Henning Glerup, Rikke H. Henriksen, Tanja Guldmann, Natalia Pedersen, Sanaz Saboori, Lone Hvid, Jens F. Dahlerup, Christian L. Hvas, Mohamad Jawhara, Karina W. Andersen, Andreas K. Pedersen, Ole H. Nielsen, Fredrik Bergenheim, Jacob B. Brodersen, Berit L. Heitmann, Thorhallur I. Halldorsson, Uffe Holmskov, Anette Bygum, Robin Christensen, Jens Kjeldsen, Torkell Ellingsen, Vibeke Andersen

**Affiliations:** ^1^The Molecular Diagnostics and Clinical Research Unit, Departement of Blood Samples, Biochemistry and Immunology, University Hospital of Southern Denmark, Aabenraa, Denmark; ^2^Section for Biostatistics and Evidence-Based Research, The Parker Institute, Bispebjerg and Frederiksberg Hospital, Copenhagen, Denmark; ^3^Department of Regional Health Research, University of Southern Denmark, Odense, Denmark; ^4^Department of Molecular Medicine, University of Southern Denmark, Odense, Denmark; ^5^Research Unit of Rheumatology, Department of Clinical Research, University of Southern Denmark, Odense University Hospital, Odense, Denmark; ^6^Department of Cancer and Inflammation Research, Odense University Hospital, Odense, Denmark; ^7^Department of Medical Gastroenterology, Odense University Hospital, Odense, Denmark; ^8^University Research Clinic for Innovative Patient Pathways, Silkeborg Regional Hospital, Silkeborg, Denmark; ^9^Department of Gastroenterology, Slagelse Hospital, Slagelse, Denmark; ^10^Department of Dermatology and Allergy Centre, Odense University Hospital, Odense, Denmark; ^11^Department of Hepatology and Gastroenterology, Aarhus University Hospital, Aarhus, Denmark; ^12^Department of Surgery, University Hospital of Southern Denmark, Aabenraa, Denmark; ^13^Department of Research and Learning, University Hospital of Southern Denmark, Aabenraa, Denmark; ^14^Department of Gastroenterology, Herlev Hospital, University of Copenhagen, Herlev, Denmark; ^15^Department of Gastroenterology, Hospital of Southwest Jutland, Esbjerg, Denmark; ^16^Research Unit for Dietary Studies, The Parker Institute, Bispebjerg and Frederiksberg Hospital, Copenhagen, Denmark; ^17^Section for General Practice, Department of Public Health, University of Copenhagen, Copenhagen, Denmark; ^18^Faculty of Food Science and Nutrition, School of Health Sciences, University of Iceland, Reykjavík, Iceland; ^19^Clinical Institute, University of Southern Denmark, Odense, Denmark; ^20^Research Unit of Medical Gastroenterology, Department of Clinical Research, University of Southern Denmark, Odense, Denmark; ^21^Open Patient Data Explorative Network, Department of Clinical Research, University of Southern, Odense, Denmark

**Keywords:** chronic inflammatory disease, biologic therapy, diet, red meat, fibre, processed meat, rheumatoid arthritis, inflammatory bowel disease

## Abstract

**Background:**

Biologic disease-modifying drugs have revolutionised the treatment of a number of chronic inflammatory diseases (CID). However, up to 60% of the patients do not have a sufficient response to treatment and there is a need for optimization of treatment strategies.

**Objective:**

To investigate if the treatment outcome of biological therapy is associated with the habitual dietary intake of fibre and red/processed meat in patients with a CID.

**Methods:**

In this multicentre prospective cohort study, we consecutively enrolled 233 adult patients with a diagnosis of Crohn's Disease, Ulcerative Colitis, Rheumatoid Arthritis (RA), Axial Spondyloarthritis, Psoriatic Arthritis and Psoriasis, for whom biologic therapy was planned, over a 3 year period. Patients with completed baseline food frequency questionnaires were stratified into a high fibre/low red and processed meat exposed group (HFLM) and an unexposed group (low fibre/high red and processed meat intake = LFHM). The primary outcome was the proportion of patients with a clinical response to biologic therapy after 14–16 weeks of treatment.

**Results:**

Of the 193 patients included in our primary analysis, 114 (59%) had a clinical response to biologic therapy. In the HFLM group (*N* = 64), 41 (64%) patients responded to treatment compared to 73 (56%) in the LFHM group (*N* = 129), but the difference was not statistically significant (OR: 1.48, 0.72–3.05). For RA patients however, HFLM diet was associated with a more likely clinical response (82% vs. 35%; OR: 9.84, 1.35–71.56).

**Conclusion:**

Habitual HFLM intake did not affect the clinical response to biological treatment across CIDs. HFLM diet in RA patients might be associated with better odds for responding to biological treatment, but this would need confirmation in a randomised trial.

**Trial registration:**

(clinicaltrials.gov), identifier [NCT03173144].

## Introduction

Crohn's disease (CD), Ulcerative Colitis (UC), Rheumatoid Arthritis (RA), Axial Spondyloarthritis (axSpA), Psoriatic Arthritis (PsA), and Psoriasis (PsO) are known as Chronic Inflammatory Diseases (CID). These CID's share pathophysiological pathways, genetics, environmental factors and pharmacological therapies (1). They are prevalent conditions, especially in Westernised, high-income countries with estimated prevalences of 0.25–1.99% (2–6) and the disease burden is predicted to rise (3, 5, 7). CIDs impact education, employability, social and interpersonal functioning negatively, thereby influencing patients' quality of life (8–10). Biologic disease-modifying drugs have revolutionised the treatment of CIDs (1). Unfortunately, up to 60% of the patients have no or only suboptimal response to biological treatment (1, 11, 12). Therefore there is an apparent need to optimise current treatment strategies (1, 13).

Aside pharmacologic treatment, many patients are interested in self-management strategies for their symptoms and often request dietary recommendations. However, sound evidence for such recommendations is lacking, hence, research within this field is imperative. Diet may have anti- or pro-inflammatory effects through its effect on the colonic microbiome which is recognised as important in CID pathogenesis (14–16), e.g., in the gut-joint axis or the gut-skin axis (14, 16), microbiome dysbiosis and abnormal intestinal barrier permeability (14, 16, 17). Short-chain fatty acids, which are products of bacterial fermentation of dietary fibres in the colon, play a crucial role in maintaining colonic epithelial integrity (18, 19). Thus, reduced intake of dietary fibre has been correlated with a thinner colonic mucus (20). Furthermore, end-products of red and processed meat fermentation in the colon have detrimental effects on colonic epithelial health (21). Therefore, a diet rich in dietary fibre and low in red/processed meat may have a protective effect on the gut and reduce systemic inflammation in CID, potentially complementing biological therapy.

The primary objective of this prospective, multicentre cohort study was to compare the proportion of CID patients achieving clinical response to biologics whilst exposed to their habitual diet high in fibre and low in red/processed meat relative to the unexposed CID patients after 14–16 weeks of treatment. Secondary outcomes included changes in health-related quality of life, physician's global assessment and C-reactive protein.

## Materials and methods

The study was approved by The Regional Committees on Health Research Ethics for Southern Denmark (S-20160124), the processing of personal data was notified to and approved by the Region of Southern Denmark and listed in the internal record (18/13682) cf. Art 30 of the EU General DATA Protection Regulation. Additionally, the study was registered at Clinical.Trials.gov (NCT03173144) and a protocol published before initiation of the study (22). A statistical analysis plan (SAP) was specified before database closure and the beginning of any statistical analyses (Supplementary File 1). Findings are reported according to the STROBE statement (23).

### Study population and setting

The study has been described previously (22). Briefly, we conducted a prospective multicentre cohort study at nine Danish clinical centres with prospectively enrolment of adult patients diagnosed with CID planned to start treatment with a biologic disease-modifying drug. Participants completed questionnaires from home and were examined in the clinics at baseline (prior to initiation of biologic therapy) and after 14–16 weeks of treatment, which is when we expect to observe a potential clinical effect. Patients who did not answer the Food Frequency Questionnaire (FFQ) administered before treatment initiation were excluded. We enrolled patients over a 3-year period from September 21st, 2017 to March 30th, 2020. Enrolment of patients was terminated before reaching the planned number of 320 patients (22) due to the Covid-19 pandemic.

Two patient associations (the Danish Colitis-Crohn's Association and the Danish Psoriasis Association) and three patient representatives diagnosed with RA were involved in developing recruitment plans as well as design of the study and communication about the study to patient members. The results were discussed with patient partners at the Department of Rheumatology at the University Hospital of Odense and an Inflammatory Bowel Disease (IBD) advisory board patient advisory board at the University Hospital of Southern Denmark—Aabenraa.

### Data collection

Study data were collected and managed using REDCap (Research Electronic Data Capture) electronic data capture tools (24, 25) hosted at OPEN (Open Patient data Explorative Network), Department of Clinical Research, University of Southern Denmark. Patients were assessed after signing the informed consent at baseline and after a period of 14–16 weeks of treatment. Physicians and study nurses were oriented individually about the study and the standardised data collection forms in REDCap by study personnel. Attending physicians completed all disease activity assessments of patients and CID specific standardised data forms on disease activity at baseline and follow-up (for details, please refer to the SAP): Harvey Bradshaw Index (HBI, CD), Mayo Clinic Score (UC), Simple Clinical Colitis Activity Index (SCCAI, UC), Disease Activity Score 28-CRP (DAS28-CRP, RA and PsA), Simplified Disease Activity Index (SDAI, RA and PsA), 46 joint count (RA), Bath Ankylosing Spondylitis Metrology Index (BASMI, axSpA), Psoriasis Area and Severity Index (PASI, PsO), 66/68 joint count (PsA) as well as a global assessment (all). Physicians or trained study nurses also collected data from medical records about diagnosis, disease localisation, disease duration, history of biological medication, concomitant medication and data on height and weight from clinical visits.

Patients reported smoking status, physical activity and dietary intake *via* an electronic questionnaire sent to them at baseline and follow-up. Patients also reported on the generic quality of life measures [the Short Form Health Survey-12 (SF-12) and the Short Health Scale] as well as CID specific quality of life measures [RA and PsA; Health Assessment Questionnaire Disability Index (HAQ-DI), PsO; Dermatology Life Quality Index (DLQI)]. Furthermore, patients reported on disease activity scores relevant for axSpA; Bath Ankylosing Spondylitis Functional Index (BASFI) and Bath Ankylosing Spondylitis Disease Activity Index (BASDAI). Details are explained in the SAP (see Supplementary File 1). Data management and study coordination were conducted at the University Hospital of Southern Denmark (Aabenraa), the Molecular Diagnostics and Clinical Research Unit.

### Dietary assessment

The FFQ used in the present study was developed and validated in relation to the 2007–2008 Danish Health Examination Survey (26, 27). It was internet-based and administered to the participants prior to initiation of biological therapy with questions covering the habitual dietary intake during the past month. The FFQ contained information from 267 different food groups. For each item, frequency of consumption was evaluated by eight categories ranging from “newer/seldom” to “twice or more per day.” In order to quantify portion sizes for main meals and other main food items, a photographic food atlas consisting of different food and meal series was included at the end of the questionnaire. However, fixed portion sizes were used for some food items with more standardised portion sizes such as fruits. The actual weight in grams for each food item was derived by multiplying the reported frequency of consumption with estimated portion sizes. Thus, total energy intake (Kj/day) and intake of different nutrients including fibres (g/day) was quantified by multiplying the amount consumed of each food item with the amount of energy or nutrient in that food according to the Danish Food Composition Tables (National Food Institute, Technical University of Denmark, https://frida.fooddata.dk/) and aggregating that contribution over all food items in the FFQ. The food group “Red/processed meat” includes beef, veal, pork, lamb, venison, charcuterie, cold cuts and entrails. The Danish Food Composition Tables are maintained and regularly updated by the National Food Institute at the Technical University of Denmark.

### Main outcomes

The primary endpoint was the proportion of participants with a clinical response to biologic therapy at the follow-up visit (14–16 weeks from baseline). The specific criteria for clinical response varied across the CID conditions (22):

- Crohn's disease: clinical remission, defined as Harvey-Bradshaw Index of 4 or less;
- Ulcerative colitis: clinical remission, defined as Mayo Clinic Score of 2 or less (with no individual sub score of >1);
- Rheumatoid arthritis: clinical response, defined as at least a 20% improvement according to the criteria of the American College of Rheumatology (ACR20) (28);
- Axial spondyloarthritis: clinical response, defined as at least a 20% improvement according to the Assessment of Spondyloarthritis International Society (ASAS20) (29);
- Psoriatic arthritis: clinical response, defined as at least a 20% improvement according to the criteria of ACR20;
- Psoriasis: clinical response, defined as at least a 75% improvement in Psoriasis Area and Severity Index (PASI 75).

Important secondary (generic) outcomes included changes from baseline to follow-up in measures of health-related quality of life (SF-12; the physical and mental component summaries, the short health scale consists of four components: symptom burden, functional status, disease-related burden and general wellbeing), C-reactive protein, and physician's global assessment. Additionally, the proportion of patients continuing biologic treatment beyond the follow-up period was a secondary outcome measure.

Other secondary non-generic outcomes listed at clinical.trials.gov included changes from baseline to follow-up in disease scores (e.g., ΔHBI score, ΔMayo Clinic score, Δtender joint count, Δswollen joint count, ΔPASI score etc., see SAP).

### Statistical analyses

We followed the prespecified statistical analysis plan (Supplementary File 1) based on the analyses outlined in the original protocol (22). The efficacy of treatment was explored in relation to the baseline dietary habits of the participants. Based on their ratio of fibre to red/processed meat intake, participants were divided into an exposed “high fibre low meat” group (=HFLM, consisting of the upper tertile of the study sample) and an unexposed “low fibre high meat” group (=LFHM, consisting of remaining participants, i.e., the lower 66.6% of the study sample). We summarised selected baseline characteristics for HFLM and LFHM by descriptive statistics. We calculated standardised differences to compare the distribution of baseline covariates between groups (30): a standardised difference above 0.5 SD-units was considered indicative of a potential (data-driven) confounder.

The independent contribution of the sub-components to the primary composite outcome were visualised in a forest plot: In the “As Observed” population (i.e., complete data for clinical response), we calculated the ORs and 95% confidence interval (95% CI) of clinical responses in the groups within each CID and pooled the estimates using random-effects meta-analysis (STATA version 16.1, the “metan” package) as we assumed normal distribution of logOR and moreover expected heterogeneity across CIDs.

For the Intention-to-Treat (ITT) population, differences in the proportions of participants with clinical responses between groups were analysed in two logistic regression models: (i) in the “crude model,” we adjusted only for CID; whereas, in the adjusted model (ii) we adjusted for CID, sex, age, and smoking status (ordinal scale: never, former, occasional and current), which were *a priori* considered potential confounding variables. Initially, clinical centre was also included as a covariate in the model but excluded in the best fitting model. Missing values were imputed by Markov Chain Monte Carlo multiple imputations using the SAS PROC MI procedure (31), assuming that the data were missing at random. We conducted five rounds of multiple imputations. For the dichotomous outcomes, we combined the estimates of the logistic regression analyses of each imputed dataset using Rubin's Rule (32). The multiple imputation datasets for continuous outcomes were combined using linear mixed effect analyses with the inclusion of patient ID as random effect and imputation as a fixed factor variable. The estimates for the continuous outcomes are reported as least-squares means with standard errors. *P* values were two-sided, with *P* < 0.05 considered potentially statistically significant. We did not explicitly adjust for multiple comparisons, however due to the implied issues of multiplicity, the secondary outcomes were interpreted with caution based on the Hochberg sequential procedure (33). These analyses where repeated when we (exploratively) analysed two secondary predictors of interest; (1) the upper tertile vs. the two lower tertiles of the study sample with regard to fibre intake [high fibre (HF) vs. low fibre (LF)] and (2) the lower tertile vs. the two upper tertiles of the study sample with regard to red/processed meat intake [low meat (LM) vs. high meat (HM)]. However, beside the previous covariates we also adjusted for red/processed meat intake in the first comparison and fibre intake in the second comparison.

To assess the predictive value of dietary exposure on clinical response on a continuous scale (i.e., independent of arbitrary tertile thresholds), we performed ROC curve analyses for the ratio of fibre to red/processed meat intake and for fibre and red/processed meat intake separately by calculating the sensitivity and specificity for each exposure variable on predicting clinical response and thereafter plotting sensitivity against 1-specificity.

To test the robustness of our results, we carried out six sensitivity analyses on the primary outcome and selected key secondary outcomes; complete case analyses (Supplementary Table S2), per protocol analysis (Supplementary Table S3), non-responder imputation analysis (Supplementary Table S4), an analysis on the subset of the study population fulfilling the original eligibility criteria (i.e., naive to biologic therapy and commencing TNF inhibitor treatment) (Supplementary Table S5), a statistical test for interaction comparing the biologic naive subgroup vs. the bio-experienced subgroup (Supplementary Figure S1) (34, 35) and an analysis with the further inclusion of BMI as covariate (Supplementary Table S8). Moreover, we carried out a propensity score analysis with full optimal matching to assess the potential confounding effect of baseline characteristic differences between the groups. Propensity score analysis was carried out in R (version 4.0.2) using the “MatchIt” package. Propensity scores were calculated by a logistic regression model including the following covariates: age, sex, CID diagnosis, smoking status, number of previous biological medication used, and disease duration. After assessing covariate balance, the marginal estimate of the effect of exposure was calculated.

## Results

We enrolled 233 participants, representing ~50% of the patients screened for eligibility (Figure 2). Many of the IBD patients screened did not enter the study. The main reasons were hospitalisation, unwillingness to participate or that treatments were initiated too fast for patients to be included in the study. 40 participants were excluded due to missing or incomplete FFQ data. Of the remaining 193 participants (representing our ITT population), 153 (81% of the study population) were naive to biologic treatment, including almost all rheumatologic patients (except four). According to the fibre/red meat ratio exposure, patients were stratified into a HFLM group (*n* = 64) and a LFHM group (*n* = 129). A total of 17 patients (8.8%) were lost to follow-up with regard to the primary outcome [HFLM: 5, 7.8% and LFHM: 12, 9.3%, *P* = 1.00 (Fischer's exact test)]. Table 1 shows the baseline characteristics; there were more women in the HFLM group (81%) compared to the LFHM group (52%). All other covariates except the intake of fibre and red/processed meat had an SMD of <0.5 SD-units i.e., they were relatively balanced between the groups.

**Table 1:** Selected baseline patient characteristics.

**Characteristics**	**Total (*N* = 193)**	**HFLM (*N* = 64)**	**N (HFLM)**	**LFHM (*N* = 129)**	**N (LFHM)**	SMD^*^
Age (years), mean (SD)	44 (15)	46 (15)	64	44 (14)	129	0.121
Women	119 (62)	52 (81)	64	67 (52)	129	0.608
Height (cm), mean (SD)	172.3 (9.1)	169.3 (7.4)	63	173.8 (9.5)	127	0.472
Weight (Kg), mean (SD)	82.3 (22.0)	76.0 (18.9)	63	85.4 (22.8)	127	0.462
BMI (Kg/m^2^), mean (SD)	27.7 (6.8)	26.5 (6.1)	62	28.2 (7.0)	127	0.269
Energy (Mj/day), mean (SD)	8.4 (3.4)	7.9 (2.8)	64	8.6 (3.7)	129	0.216
Fibre (g/day), mean (SD)	18.1 (8.2)	21.6 (8.6)	64	16.3 (7.4)	129	0.608
Red/processed meat (g/day), mean (SD)	95.6 (84.1)	51.3 (22.9)	64	117.5 (94.3)	129	0.608
**Smoking status**			64		129	0.356
Non-smoker	83 (43)	36 (56)	64	47 (36)	129	0.403
Former	69 (36)	21 (33)	64	48 (37)	129	0.074
Occasionally	4 (2)	1 (2)	64	3 (2)	129	0.000
Daily	37 (19)	6 (9)	64	31 (24)	129	0.362
**CID diagnosis**			64		129	0.211
Crohn's disease	55 (28)	15 (23)	64	40 (31)	129	0.156
Ulcerative colitis	40 (21)	17 (27)	64	23 (18)	129	0.197
Rheumatoid arthritis	37 (19)	17 (27)	64	20 (16)	129	0.037
Axial spondyloarthropathy	28 (15)	8 (13)	64	20 (16)	129	0.065
Psoriatic arthritis	24 (12)	6 (9)	64	18 (14)	129	0.106
Psoriasis	9 (5)	1 (2)	64	8 (6)	129	0.166
Disease duration (years), median (IQR)	6 (1–13)	4 (1–10)	64	7 (2–13)	129	0.227
**No of previous biologics used**			63		126	0.027
0	153 (81)	53 (84)	63	100 (79)	126	0.117
≥1	36 (19)	10 (16)	63	26 (21)	126	0.000
**Medication**
None	17 (9)	8 (13)	64	9 (7)	129	0.166
NSAID, daily use	21 (11)	7 (11)	64	14 (11)	129	0.000
Corticosteroids	57 (30)	15 (23)	64	42 (33)	129	0.180
Immunomodulators	72 (37)	28 (44)	64	44 (34)	129	0.192
5-ASA/SASP	53 (27)	17 (27)	64	36 (28)	129	0.000
Antibiotics	3 (2)	0 (0)	64	3 (2)	129	0.092
Hydroxychloroquin	21 (11)	11 (17)	64	10 (8)	129	0.296
Leflunomid	7 (4)	1 (2)	64	6 (5)	129	0.121
**Other outcome measures, median (IQR)**						
SF-12 PCS (0–100)	41.67 (38.58–45.21)	41.98 (39.33–46.00)	64	41.53 (38.19–44.69)	129	0.227
SF-12 MCS (0–100)	47.45 (42.15–53.02)	47.92 (42.24–52.44)	64	47.45 (41.80–53.08)	129	0.006
Symptom burden (0–100)	62.5 (50.0–75.0)	67.5 (54.5–77.0)	64	62.0 (48.0–74.0)	128	0.227
Functional status (0–100)	62.5 (35.0–76.5)	63.5 (36.0–80.5)	64	61.0 (31.5–75.0)	128	0.233
Disease-related burden (0–100)	66.5 (43.0–80.0)	67.0 (46.5–82.5)	64	65.5 (41.0–79.5)	128	0.080
General wellbeing (0–100)	52.0 (31.0–71.0)	56.5 (34.0–73.5)	64	51.0 (31.0–70.0)	128	0.156
Phys. global assessment (0–100 VAS)	50.0 (31.0–67.0)	55.0 (35.0–68.0)	43	45.0 (30.5–66.0)	80	0.205
CRP, mg/L	3.9 (1.7–11.0)	3.9 (1.5–9.6)	57	3.9 (1.7–12.0)	107	0.021

Standardised mean differences (SMD) was calculated based on P-values derived from two-sample t-test for continuous variables and from Fischer's exact test for binary and categorical variables; a standardised difference value of 0.000 interprets into no difference between the groups.

* A SMD between HFLM and LFHM above 0.5 SD-units will be evaluated as a potential (data driven) confounding variable. HFLM, high fibre/low meat (exposed); LFHM, low fibre/High meat; CID, chronic inflammatory disease; IQR, interquartile range; NSAID, non-steroidal anti-inflammatory drugs; 5-ASA/SASP, 5-aminosalicyclic acid/sulfasalazine; PROs, patient reported outcomes; HRQoL, health related quality of life; SF-12, 12-item short form survey; PCS, physical component summary; MCS, mental component summary; Phys; physician; VAS, visual analogue scale; CRP, C-reactive protein. Symptom burden, functional status, disease-related burden and general wellbeing are components of the short health scale.

Values are numbers (percentages) unless otherwise specified.

**Figure 1:**
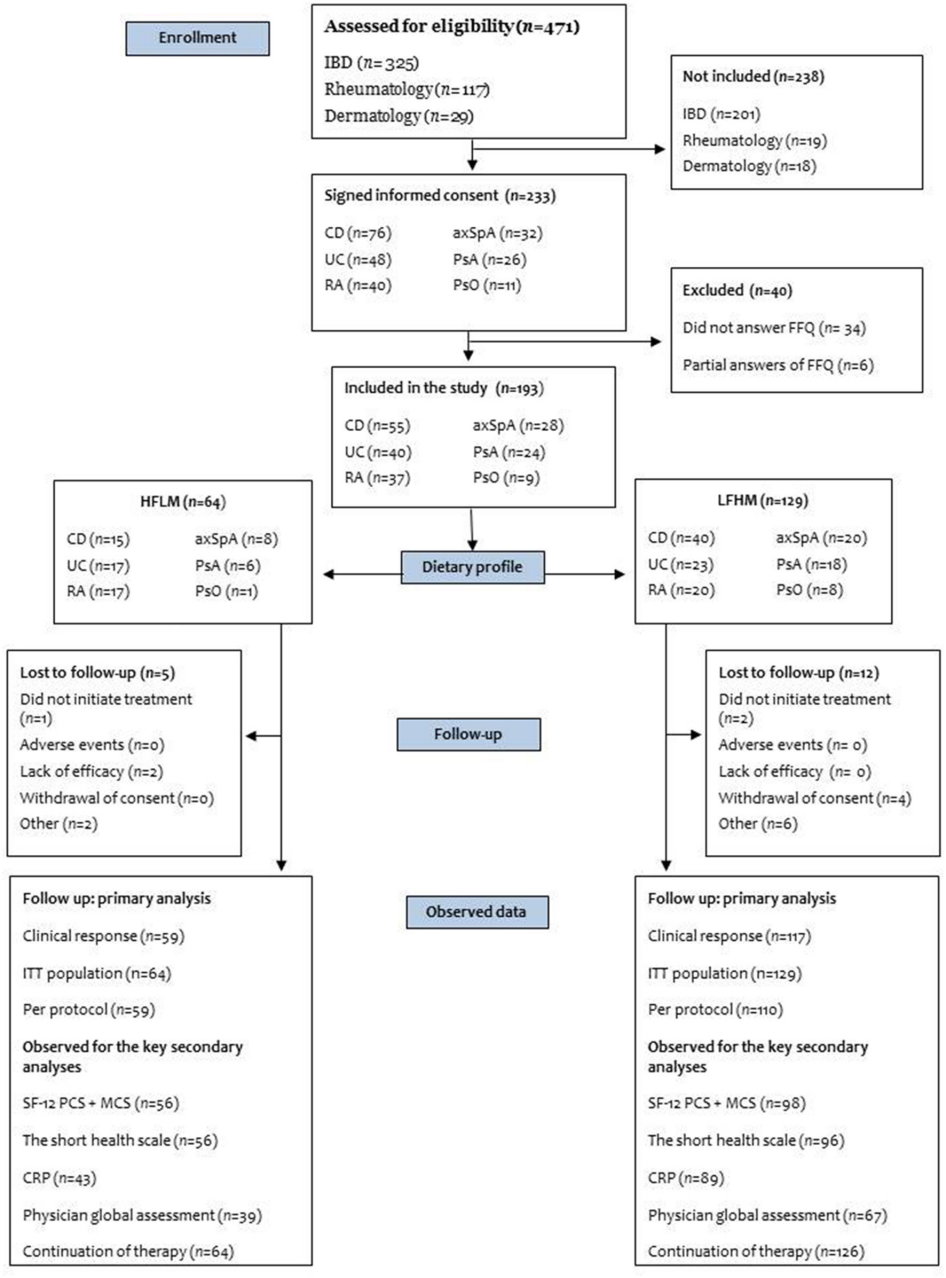
Flow chart of the enrolment of participants. Number of Chronic Inflammatory Disease patients who were screened, included in the study and included in the analysis. Patients screened for eligibility were not explicitly recorded in the study. For CD, UC, and PsO we estimated a number based on the mean inclusion rate from two clinics that kept a pre-screening log. For RA, axSpA, and PsA, the number is not an estimate but the total number of patients that initiated biologic treatment in the clinic during enrolment. IBD, Inflammatory bowel disease (Crohn's disease and Ulcerative colitis); CD, Crohn's disease; UC, Ulcerative colitis; RA, Rheumatoid arthritis; axSpA, Axial spondyloarthritis; PsA, Psoriatic arthritis; PsO, Psoriasis; FFQ, Food Frequency Questionnaire; HFLM, high fibre/low meat; LFHM, low fibre/high meat; ITT, intention-to-treat; SF-12 PCS + MCS, short form health survey the physical and mental component summary, The short health scale includes four health dimensions (symptom burden, functional status, disease-related burden and general wellbeing); CRP, C-reactive protein.

A total of 108 patients out of the 176 (61.4%) from whom we had data on regarding clinical response (i.e., the “As observed” population) responded to the biologics at follow-up according to our specified criteria for clinical response. On the “As observed population,” we included the absolute number of clinical response (events) for HFLM and LFHM in a meta-analysis of the included CIDs (Figure 1). There was no difference between groups in the primary (combined) analysis (OR 1.14, 95% CI: 0.41, 3.15) but the *I*^2^ of 50.7% indicated moderate to substantial heterogeneity across CIDs. When looking at the individual strata, the OR for RA patients was in favour of the HFLM group (OR 8.56, 95% CI: 1.74–42.17). Based on logistic regression models on the ITT population, 64% responded to biologics in the HFLM exposed group compared to 56% in the LFHM group (Table 2, OR 1.39, 95% CI: 0.72–2.69). After adjusting for sex, age, CID, and smoking status, the OR was 1.48 (Table 2, 0.72–3.05). For RA patients, the OR when comparing HFLM to LFHM in the adjusted model was 9.84 (1.35–71.56). Changes in health-related quality of life (SF-12 PCS, SF-12 MCS, and the Short Health Scale), physician's global assessment and C-reactive protein were similar between groups (Table 2). Likewise, the proportion of patients continuing biologic treatment beyond the 14–16 weeks was similar between groups.

**Figure 2:**
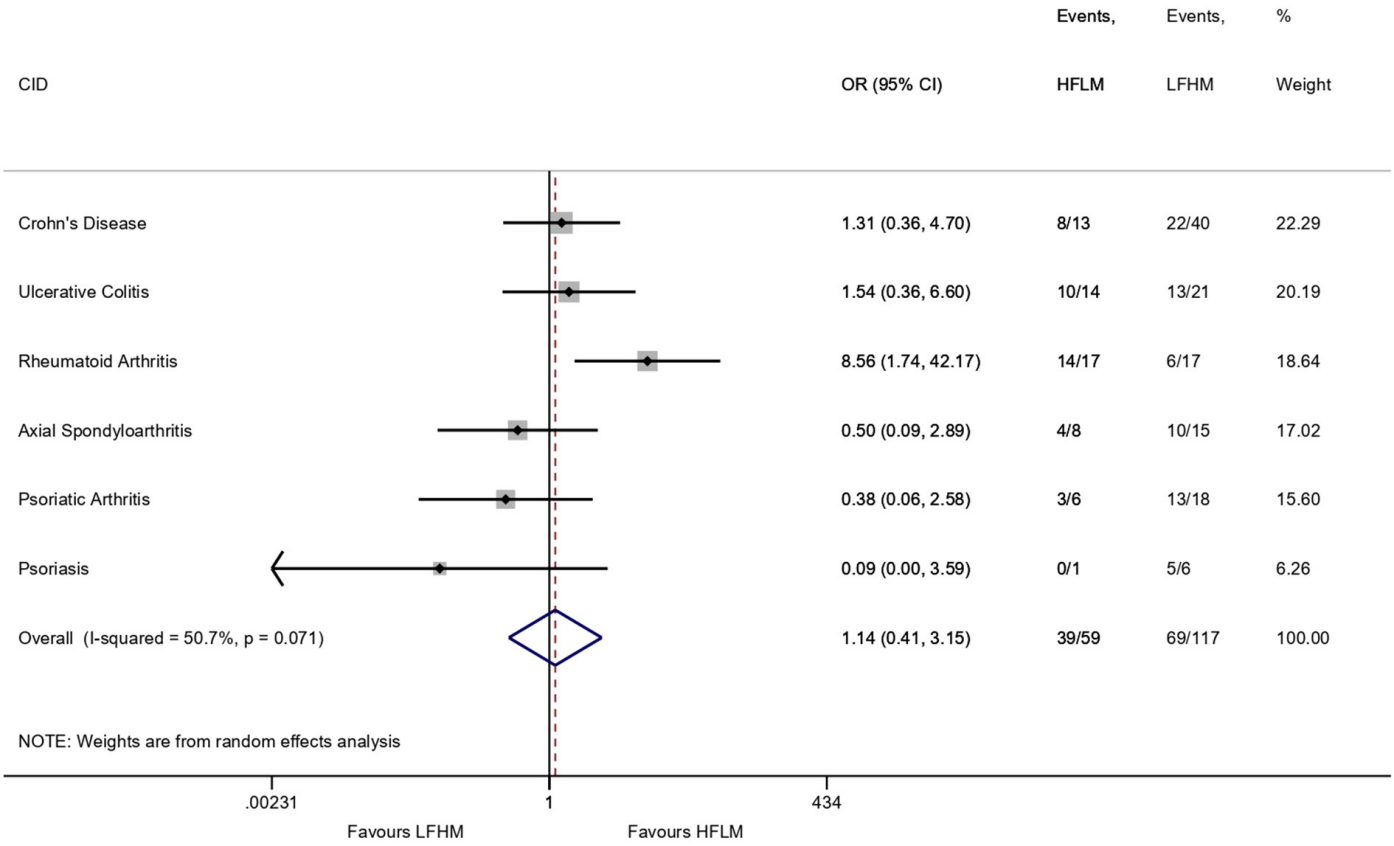
Meta-analysis (random effects model) of the included Chronic Inflammatory Diseases (CIDs) on the “As Observed” population comparing clinical response to biologics in patients with a high intake of fibre and low intake of red/processed meat (HFLM) vs. patients with a low intake of fibre and high intake of red/processed meat (LFHM). The horizontal lines represent the odds ratio (OR) ± 95% confidence interval. Event = clinical response according to the specified criteria for each CID, i.e., the number shows how many out of the total number of participants in the group, that have had a clinical response. The “As Observed” populations means patients with complete data for clinical response (i.e., 17 patients are excluded).

**Table 2:** Primary and key secondary outcomes.

**Outcome**	**Crude model** ^a^				**Adjusted model** ^b^			
	**HFLM (*N* = 64)**	**LFHM (*N* = 129)**	**Contrast (95% CI)**	***P*** -value^*^	**HFLM**	**LFHM**	**Contrast (95% CI)**	***P*** -value^*^
**Primary outcome (composite)**								
Clinical response	41 (64)	73 (56)	1.39 (0.72; 2.69)	0.329	–	–	1.48 (0.72; 3.05)	0.285
**Sub-components**								
Crohn's disease, HBI ≤ 4	8 (53)	22 (55)	0.94 (0.28; 3.06)	n.a.	–	–	0.68 (0.18; 2.65)	n.a.
Ulcerative colitis, mayo ≤ 2	12 (69)	14 (61)	1.48 (0.33; 6.61)	n.a.	–	–	2.62 (0.40; 17.22)	n.a.
Rheumatoid arthritis, ACR20 response	14 (82)	7 (35)	9.50 (1.95; 46.16)	n.a.	–	–	9.84 (1.35; 71.56)	n.a.
Axial spondyloarthritis, ASAS20 response	4 (50)	11 (56)	0.78 (0.14; 4.51)	n.a.	–	–	0.59 (0.02; 14.56)	n.a.
Psoriatic arthritis, ACR20 response	3 (50)	13 (72)	0.38 (0.06; 2.58)	n.a.	–	–	0.19 (0.02; 2.23)	n.a.
Psoriasis, PASI75 response	0 (0)	6 (75)	n.a.	n.a.	–	–	n.a.	n.a.
**Key secondary outcomes**								
**Health-related quality of life**								
ΔSF-12 PCS (0–100)	−1.68	−1.24	−0.44 (−1.62; 0.74)	0.464	−1.76	−1.21	−0.55 (−1.79; 0.69)	0.383
ΔSF-12 MCS (0–100)	1.75	0.51	1.24 (−0.28; 2.76)	0.109	1.57	0.60	0.98 (−0.62; 2.57)	0.23
ΔSymptom burden (0–100)	−27.80	−22.58	−5.22 (−12.21; 1.77)	0.143	−29.71	−21.63	−8.08 (−15.24; −0.92)	0.027
ΔFunctional status (0–100)	−25.83	−18.94	−6.89 (−14.26; 0.48)	0.067	−27.32	−18.2	−9.12 (−16.67; −1.56)	0.018
ΔDisease-related burden (0–100)	−22.59	−21.37	−1.22 (−8.79; 6.35)	0.752	−23.11	−21.12	−1.99 (−9.95; 5.98)	0.625
ΔGeneral wellbeing (0–100)	−19.15	−16.99	−2.16 (−8.87; 4.55)	0.528	−20.6	−16.27	−4.33 (−11.27; 2.61)	0.221
ΔCRP (mg/L)	−1.75	−1.46	−0.29 (−8.10; 7.52)	0.942	−1.4	−1.64	0.24 (−7.96; 8.43)	0.955
ΔPhysicians global assessment (0–100 mm VAS)	−40.11	−42.62	2.51 (−4.70; 9.72)	0.495	−38.51	−43.42	4.90 (−2.56; 12.37)	0.198
Continuation of treatment	56 (88)	105 (82)	1.70 (0.69; 4.17)	0.248	–	–	1.96 (0.75; 5.14)	0.171
**Safety/harms**								
Withdrawals	2 (3)	5 (4)	n.a.	n.a.	–	–	n.a.	n.a.
Discontinuation due to adverse events	1 (2)	8 (6)	n.a.	n.a.	–	–	n.a.	n.a.
Serious adverse events (SAEs)	1 (2)	3 (2)	n.a.	n.a.	–	–	n.a.	n.a.

Differences in the proportions of participants with clinical responses between groups were analysed in two logistic regression models:

a the “crude model” were adjusted only for CID,

b the adjusted model were adjusted for CID, sex, age and smoking status (ordinal scale: never, former, occasional and current). Missing values were imputed by Markov Chain Monte Carlo multiple imputations assuming that the data were missing at random. Five rounds of multiple imputations were conducted; for the dichotomous outcomes, we combined the estimates of the logistic regression analyses of each imputed dataset using Rubin's Rule while the datasets for continuous outcomes were combined using linear mixed effect analyses with the inclusion of patient ID as random effect and imputation as a fixed factor variable.

* As outlined in the statistical analysis plan, we pre-specified not to report P-values for the sub-components of the primary outcome (i.e., the individual CIDs) due to the low number of patients and therefore “n.a.” is reported. The key secondary outcomes are interpreted based on the Hochberg sequential procedure. HFLM, high fibre/low meat group (the upper tertile of the study sample with regard to fibre to red/processed meat ratio); LFHM, low fibre/high meat (the two lower tertiles of the study sample with regard to the fibre to red/processed meat ratio); CI, confidence interval; HBI, Harvey Bradshaw index; Mayo, mayo clinic score, ACR20, 20% improvement according to the criteria of the American College of Rheumatology; ASAS20, 20% improvement according to assessment of Spondyloarthritis International Society; PASI75, 75% improvement in the Psoriasis Area and Severity Index; CRP, C-reactive protein; VAS, visual analogue scale; SF-12, 12-item short form survey; PCS, physical component summary; MCS, mental component summary. Symptom burden, functional status, disease-related burden and general wellbeing are components of the short health scale.

Values are numbers (percentages) with odds ratios (95%CI) for dichotomous outcomes and least squares mean with differences (95%CI) for the continuous variables.

The results of the sensitivity analyses (Supplementary Tables S2–S5, S8 and Supplementary Figure S1) are essentially in agreement with the primary analysis. However, in Supplementary Table S5, when analysing the subset of the study population that were naive to biological treatment and treated with a TNF inhibitor, the OR of clinical response was 2.61 higher in the HFLM group (1.09–6.18). Thus, we carried out a subgroup analysis to test for interaction between naive and bio-experienced patients, but found no evidence to support a different impact of the diet groups on clinical response, with an estimated interaction effect (ratio of odds ratio) of 1.58 (0.74–3.35, *P* = 0.1). The propensity score analysis was also in agreement with the primary analysis (results not shown).

Finally, we investigated if fibre intake and red/processed meat where independently associated with clinical response in explorative analyses. There was no significant difference between the groups in either comparison (Supplementary Tables S6, S7).

**Figure 3:**
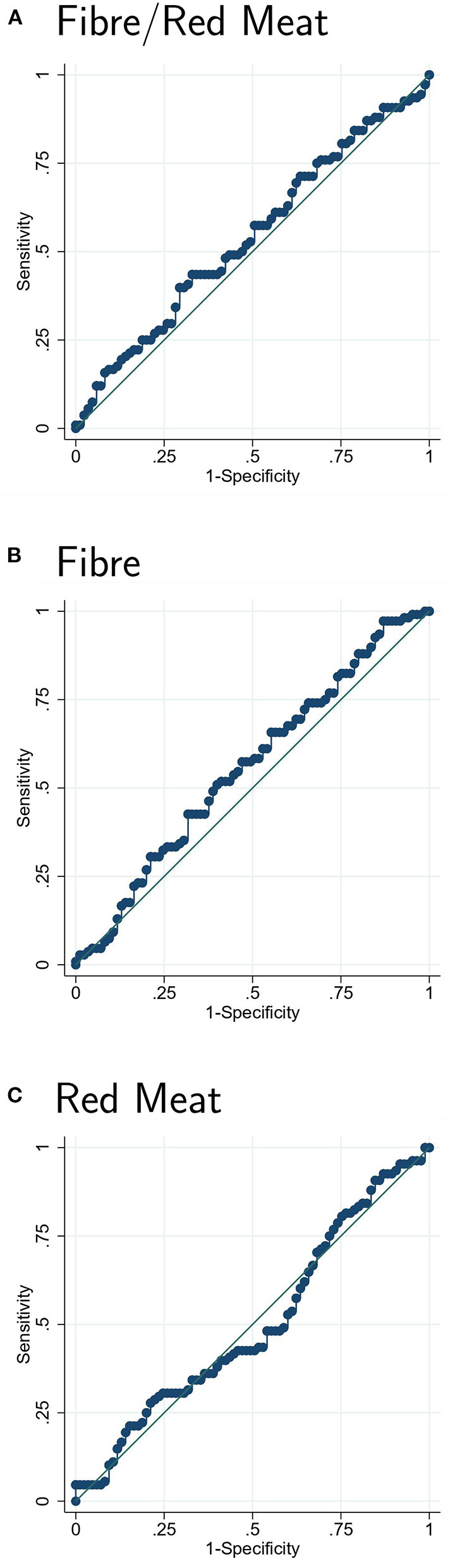
Receiver operating characteristics curve analysis for the predictive value of the ratio of fibre and red/processed meat intake **(A)**, fibre intake **(B)** and red/processed meat intake **(C)** on clinical response. The ROC curves were constructed by calculating the sensitivity and specificity for prediction of clinical response for **(A)** the ratio of fibre to red/processed meat intake, **(B)** fibre intake, and **(C)** red/processed meat intake. Thereafter the sensitivity was plotted against 1-specificity. ROC, receiver operating characteristics.

In order to fully explore whether other thresholds of fibre and meat intake could change the prognostic utility of these measures, Figure 3 represents the ROC curves which supports the finding of no association between fibre and meat intake and clinical response. There was no predictive value of the diet on clinical response for neither the ratio or the isolated intake of fibre and red/processed meat as all three curves overlap the reference line (Figure 3). Other secondary outcomes are presented in Supplementary Table S1.

## Discussion

### Principal findings

Our study evaluated if a habitual high intake of fibre and low intake of red/processed meat vs. low intake of fibre and high intake of red/processed meat was associated with clinical response to biological treatment in prospectively enrolled patients with one of six diagnoses of CID. There was no significant difference in the proportion of patients with a clinical response between groups across the CID conditions or in any of the secondary outcomes. However, our results indicate that a high habitual dietary intake of fibre and low intake of red/processed meat can complement the biologic treatment for RA patients as the odds of clinical response were almost 10 times higher in the HFLM group compared to LFHM.

### Comparison with other studies

It has been suggested that the high prevalence of CIDs in Westernised countries relates to dietary habits and lifestyle. While dietary habits may be relevant in the development (and therefore prevention) of CIDs, diet as a supporting factor for pharmacologic treatment has not received much attention. Nevertheless, a supporting effect of diet on pharmacologic treatment is possible. For instance, a meta-analysis found that enteral nutrition therapy in combination with infliximab therapy increases the efficacy for CD (36). Also, an interventional but uncontrolled study found a remission rate of 100% among newly diagnosed CD patients on infliximab (TNFi) therapy in combination with a semi-vegetarian (high fibre) diet (37).

High consumption of red/processed meat and low consumption of dietary fibres is typical of a Westernised diet, while the opposite is characteristic for a Mediterranean Diet (38). The Mediterranean diet is considered a model for healthy eating, and adherence to it is associated with reduced risk of overall mortality, occurrence of chronic diseases (39) and symptomatic improvement of RA (40). Nevertheless, the diet of the HFLM group does not represent a healthy eating pattern in terms of fibre and red/processed meat intake per se, but rather a healthier (or less unhealthy) diet in contrast to the LFHM group. The estimated daily intake of fibre in the HFLM group of 22 g/day is below the recommendations from most countries of 25–35 g/day (41) but in line with the estimated average intake of dietary fibre for Danes (22 g/day) (42) and Europeans (men: 18–24 g/day, women: 16–20 g/day) (41). On the other hand, the average intake of red/processed meat in HFLM was 51 g/day (LFHM: 118 g/day), which is above the EAT-*Lancet* Commission's proposal of a healthy dietary pattern (14 g/day) (43). However, the intake corresponds well with estimates of mean intakes of red and processed meat among European countries (71–93 g/day) (44). Yet, the intake in our study is low in comparison with a report of the Danes dietary habits from 2011–2013 (134 g/day) (42).

An explanation for the potential benefit of the HFLM diet for RA patients, that we observe in our study in contrast to the other CIDs, may lie in the different positions of the CIDs on the immunological disease continuum extending from “pure” autoinflammatory diseases to “pure” autoimmune diseases (45–47). Although there are mixed autoinflammatory and autoimmune patterns among the CIDs, RA is classified as an autoimmune disease in the classical sense whereas the other CIDs are more classified as autoinflammatory (45, 48). Thus, it can be speculated that the adaptive immune system may be more susceptible to dietary influences. Another explanation may be the fact that almost all RA patients in our study were naive to biologics. Studies have found that rheumatic patients naive to TNFi are more likely to respond to the treatment compared to biologic experienced patients (49–51). The significant differences between the groups among the RA patients in the primary analysis suggests that this likelihood of a positive response to treatment is emphasised when adding dietary habits to the equation. However, this was not the case for CIDs as a group as we found no evidence to support a different impact of the diet groups on clinical response when comparing biologic naive with biologic-experienced patients. We also looked into whether there were large differences in fibre and meat intake across the CIDs that could perhaps be part of the explanation (see Supplementary Table S9). However, the RA patients did not stand out as an extreme.

### Strengths and limitations of the study

Our study is subject to certain limitations inherent in its design. Measurement of the exposure is connected to some imprecision as it is based on FFQs evaluating the past month before treatment initiation. The FFQ is a self-reporting assessment instrument and is therefore prone to measurement error by risk of recall bias and under- or over-reporting of dietary intake. This imprecision is likely to bias the results towards unity (52). Another limitation of our study is the limited number of participants. The wide 95% confidence interval of our primary outcome indicates a risk for type 2 errors, i.e., we cannot conclude that there truly is no difference between the groups across the CID diagnoses because of insufficient power. Furthermore, perhaps the two diet groups were not sufficiently separated (i.e., indifferent) in relation to exposure and it would be more correct to name the LFHM group “non-high fibre/ non-low meat.” If we had the power, potential differences would be more obvious if we compared the upper tertile with the lower tertile as the middle tertile may blur the contrast.

Dietary assessment (i.e., the FFQ) was only carried out at baseline. Thus, our analyses are based on the assumption that the habitual dietary intake will not change over time, but this may not be accurate for especially CD and UC. It is known that IBD patients eat differently during active and inactive disease (53), thus, the dietary intake for this particular group may change during the treatment period. Nevertheless, specifically fibre intake has been observed to be the same in IBD patients with active and inactive disease (53), although lower compared to healthy controls (53, 54). Thus, the intake of fibres may be relatively stable. However, if the intake of fibre and red/processed meat have changed during the study period for the study participants, there is a risk of misclassification bias, which can have unpredictable results as it can either increase or decrease the association. Furthermore, intake of fibres and red/processed meat may very well be associated with intake of other macro- and micro nutrients and foods. Thus, the diet other than fibre and meat intake will probably vary considerably between HFLM and LFHM (55), which may be partly responsible for the observed effect or lack of.

The eligibility criteria for the study were changed after protocol development and initiation of the study (i.e., bio-experienced patients and patients initiating other biologics than TNFi were also made eligible), which can sometimes create bias. However, the amendment was not made based on looking at the data but to increase the sample size. If anything, the heterogeneity of the study sample, which was increased due to the change, may have weakened a possible association. Indeed, a sensitivity analysis of the subgroup fulfilling the original eligibility criteria showed differences between the diet groups.

The study also has several strengths, including its prospective study design and rigorous study protocol (22). In addition, it is a real-life pragmatic cohort that strengthens the external validity of the study. Furthermore, participants were unaware of the specific exposure (fibre and red/processed meat intake) being studied. Finally, our results were robust to multiple sensitivity analyses, including applying an alternative approach (propensity score analysis), except for the above mentioned which found a difference between groups.

## Conclusion

In conclusion, we found no difference in clinical response to treatment with biologics among CID patients exposed to a habitual HFLM diet compared to a LFHM diet. Yet, our results suggest a potential benefit of the HFLM diet among RA patient, however, due to the imprecision of the exposure, the observational study design, and because the analysis is based on a relatively small number of patients, more research on the subject is needed—preferably from interventional studies.

## Data availability statement

The raw data supporting the conclusions of this article will be made available upon request pending application and approval, contact: va@rsyd.dk.

## Ethics statement

The studies involving human participants were reviewed and approved by the Regional Committees on Health Research Ethics for Southern Denmark, Vejle, Denmark. The patients/participants provided their written informed consent to participate in this study.

## Author contributions

VA and RC conceptualised the study and drafted the study protocol. SSø, HM, AN, HG, LH, JD, MJ, KA, ON, JB, BH, UH, AB, TE, JK, RC, and VA contributed to protocol development and methodology. VA and SO led the funding acquisition. HM, AB, HG, RH, TG, NP, SSa, LH, JD, CH, MJ, KA, ON, FB, JB, AB, TE, JK, SO, and SSø, participated in recruitment of patients and data collection. KA, MJ, SSø, and SO developed the data collection tool in REDCap. SSø, VA, and SO led the study co-ordination and VA the project administration. SO and RC drafted the statistical analysis plan. SO, AP, and RC performed the statistical analyses. TH analysed the FFQ. SO and RC had full access to all the data in the study and take responsibility for the integrity of the data and the accuracy of the data analysis. SO drafted the manuscript and attests that all listed authors meet authorship criteria and that no others meeting the criteria have been omitted. VA, RC, TE, JK, and SO are guarantors of the study. All authors contributed to and approved the statistical analysis plan and critically revised the manuscript for scientific content and approved the final version of the manuscript.

## Funding

This project has received funding from the European Union's Horizon 2020 research and Innovation programme under grant agreement No. 733100 (https://www.syscid.eu/). The Parker Institute, Bispebjerg and Frederiksberg Hospital is supported by a core grant from the Oak Foundation (OCAY-18-774-OFIL). The project has furthermore received funding from IBD-Care (Improved diagnosis and treatment in the Region of Southern Denmark, 17/18561), the Region of Southern Denmark, University Hospital of Southern Denmark and the memorial fund of Knud and Edith Eriksen.

## Conflict of interest

Author CH has received speaker fee from Takeda Pharma and Tillotts Pharma (unrelated to the present work). The remaining authors declare that the research was conducted in the absence of any commercial or financial relationships that could be construed as a potential conflict of interest.

## Publisher's note

All claims expressed in this article are solely those of the authors and do not necessarily represent those of their affiliated organizations, or those of the publisher, the editors and the reviewers. Any product that may be evaluated in this article, or claim that may be made by its manufacturer, is not guaranteed or endorsed by the publisher.
