# Erratum: Impact of fibre and red/processed meat intake on treatment outcomes among patients with chronic inflammatory diseases initiating biological therapy: A prospective cohort study

**DOI:** 10.3389/fnut.2022.1091846

**Published:** 2022-12-07

**Authors:** Frontiers Production Office

**Affiliations:** Frontiers Media SA, Lausanne, Switzerland

**Keywords:** chronic inflammatory disease, biologic therapy, diet, red meat, fibre, processed meat, rheumatoid arthritis, inflammatory bowel disease

Due to a production error, there was a mistake in the legend for Figure 1 as published. The legend for Figure 2 was incorrectly used in its place. The correct legend appears below.

Flow chart of the enrolment of participants. Number of Chronic Inflammatory Disease patients who were screened, included in the study and included in the analysis. Patients screened for eligibility were not explicitly recorded in the study. For CD, UC, and PsO we estimated a number based on the mean inclusion rate from two clinics that kept a pre-screening log. For RA, axSpA, and PsA, the number is not an estimate but the total number of patients that initiated biologic treatment in the clinic during enrolment. IBD, Inflammatory bowel disease (Crohn's disease and Ulcerative colitis); CD, Crohn's disease; UC, Ulcerative colitis; RA, Rheumatoid arthritis; axSpA, Axial spondyloarthritis; PsA, Psoriatic arthritis; PsO, Psoriasis; FFQ, Food Frequency Questionnaire; HFLM, high fibre/low meat; LFHM, low fibre/high meat; ITT, intention-to-treat; SF-12 PCS + MCS, short form health survey the physical and mental component summary, The short health scale includes four health dimensions (symptom burden, functional status, disease-related burden and general wellbeing); CRP, C-reactive protein.

Due to a production error, there was a mistake in the legend for Figure 2 as published. The legend for Figure 1 was incorrectly used in its place. The correct legend appears below.

Meta-analysis (random effects model) of the included Chronic Inflammatory Diseases (CIDs) on the “As Observed” population comparing clinical response to biologics in patients with a high intake of fibre and low intake of red/processed meat (HFLM) vs. patients with a low intake of fibre and high intake of red/processed meat (LFHM). The horizontal lines represent the odds ratio (OR) ± 95% confidence interval. Event = clinical response according to the specified criteria for each CID, i.e., the number shows how many out of the total number of participants in the group, that have had a clinical response. The “As Observed” populations means patients with complete data for clinical response (i.e., 17 patients are excluded).

The publisher apologizes for this mistake. The original version of this article has been updated.

## Publisher's note

All claims expressed in this article are solely those of the authors and do not necessarily represent those of their affiliated organizations, or those of the publisher, the editors and the reviewers. Any product that may be evaluated in this article, or claim that may be made by its manufacturer, is not guaranteed or endorsed by the publisher.

