# Supplementary material for: Impact of fibre and red/processed meat intake on treatment outcomes among patients with chronic inflammatory diseases initiating biological therapy: A prospective cohort study
Source: Front Nutr. 2022 Oct 13;9:985732. doi: 10.3389/fnut.2022.985732 (PMC9609158; doi:10.3389/fnut.2022.985732)
Supplement: Supplementary file 1 [file Data_Sheet_1.pdf]

# Statistical Analysis Plan (SAP)

## 1. Administrative information

### 1.1 Title, registration and version

| | |
|---|---|
| Full study title | Impact of red and processed meat and fiber intake on treatment outcomes among patients with chronic inflammatory diseases: a prospective cohort study of prognostic factors and personalized medicine. |
| Acronym | BELIEVE |
| Clinicaltrials.gov number | NCT03173144 https://clinicaltrials.gov/ct2/results?cond=&term=NCT03173144 |
| Ethics Committee number | S-20160124 |
| The Danish Data Protecting Agency number | 2008-58-035 |
| A published protocol is available at BMJ OPEN: | https://bmjopen.bmj.com/content/8/2/e018166.long |
| SAP version | 1.0 |
| SAP date | 2020-09-02 |



CONFIDENTIAL

## 1.2. Roles and responsibility

**Statistical analyst** Silja Hvid Overgaard[1,2], MSc, PhD fellow

[1]Focused Research Unit for Molecular Diagnostics and Clinical Research, IRS-Center Sonderjylland, Hospital Sønderjylland, Aabenraa, Denmark & [2]Musculoskeletal Statistics Unit, the Parker Institute, Bispebjerg and Frederiksberg Hospital, University of Copenhagen, Copenhagen, Denmark.

**Statistical advisor** Robin Christensen[1,2], MSc, Professor (Biostatistics & Clinical Epidemiology)

[1]Musculoskeletal Statistics Unit, the Parker Institute, Bispebjerg and Frederiksberg Hospital, University of Copenhagen, Copenhagen & [2]Research Unit of Rheumatology, Department of Clinical Research, University of Southern Denmark, Odense University Hospital, Denmark.

**Principle investigator** Vibeke Andersen[1,2,3], MD, Professor (Gastroenterologist)

[1]Focused Research Unit for Molecular Diagnostics and Clinical Research, IRS-Center Sonderjylland, Hospital Sønderjylland, Aabenraa, Denmark & [2]Institute of Molecular Medicine, University of Southern Denmark, Odense, Denmark & [3]OPEN Explorative Network, University of Southern Denmark, Odense, Denmark.

**Collaborators/coming manuscript authors**: Silja Hvid Overgaard, Signe Bek Sørensen, Heidi Lausten Munk, Anders Bathum Nexøe, Henning Glerup, Rikke Holm Henriksen, Tanja Guldmann, Natalia Pedersen, Sanaz Saboori, Lone Hvid, Jens Frederik Dahlerup, Christian Lodberg Hvas, Karina Winther Andersen, Mohamad Jawhara, Ole Haagen Nielsen, Fredrik Bergenheim, Jacob Brodersen, Berit L. Heitmann, Thorhallur Ingi Halldorsson, Uffe Holmskov, Anette Bygum, Torkell Ellingsen*, Jens Kjeldsen*, Robin Christensen*, Vibeke Andersen*.

* These authors share last authorship.

### 1.3. Signatures:

Date: 03-09-2020 Signature: [signature]

Silja Hvid Overgaard, MSc, PhD fellow

Date: Sep. 2/2020 Signature: [signature]

Robin Christensen, MSc, PhD, Professor (Biostatistics & Clinical Epidemiology)

Vibeke Andersen

Digitalt signeret af Vibeke Andersen
Dato: 2020.09.02 12:06:59 +02'00'

Date: Signature:

Vibeke Andersen, MD, PhD, Professor (Gastroenterologist)



CONFIDENTIAL

# 2. Introduction

## 2.1. Purpose of the Statistical Analysis Plan

The purpose of the SAP is to give a detailed elaboration of the statistical analysis planned to be executed on primary and secondary outcomes etc. corresponding to the primary and key secondary objectives in the BELIEVE Cohort study [1]. This SAP was developed after the study protocol was registered and published [2] and before database closure and conduct of any statistical analyses. Deviations from the pre-specified study protocol are summarized and explained in the SAP (section 4.2.).

Reporting of the study will follow the STROBE- (Strengthening the Reporting of Observational Studies in Epidemiology) and TRIPOD-guidelines (Transparent Reporting of a multivariable prediction model for Individual Prognosis Or Diagnosis), adhering to the guidelines for reporting of cohort studies [3, 4].

## 2.2. Background and rationale

Chronic inflammatory diseases (CID) are a diverse set of immunological diseases that include diseases of the gastrointestinal tract (Crohn's disease [CD] and ulcerative colitis [UC]), musculoskeletal and connective tissue (rheumatoid arthritis [RA], axial spondyloarthropathy [axSpA] and psoriatic arthritis [PsA]) and the skin (psoriasis [PsO]). CIDs are recurring lifelong illnesses of potentially early onset that can substantially affect the life quality of patients and their families [5-7]. Furthermore, they are prevalent diseases and the disease burden is predicted to rise [8-11]. CIDs pose a heavy burden on the community due to direct health-related costs (largely influenced by providing biological medications[11]) as well as indirect costs associated with patient's reduced productivity and sick-leave. CIDs are frequently treated with biologic drugs that target pro-inflammatory cytokines, e.g., tumor necrosis factor (TNF). However, up to 60% of the patients do not benefit from such biological therapy [11-13].

The increasing incidence of chronic inflammatory diseases in the Western world has prompted research into the potential impact of lifestyle (smoking, physical activity, urban living etc.), and many have been associated with risk of developing CIDs, including diet [14-19]. Accordingly, many patients ask their healthcare professionals for lifestyle recommendations that can influence the effectiveness of treatment. However, in a recent systematic review examining the impact of diet on TNF inhibitor (TNFi) response, it was concluded that the evidence is scarce and more research is needed [20]. Thus, we commenced on this prospective cohort study to identify dietary factors that support optimal treatment outcomes of biologic therapy. Specifically, as specified in the published protocol [2], we wanted to explore the impact of intake of fibers and red/processed meat on treatment outcomes.

## 2.3. Aim

The overall aim is to explore the prognostic value of dietary patterns on response to treatment in patients with CIDs initiating treatment with a biological agent.

## 2.4. Hypotheses

We hypothesize that a diet high in fiber *AND* low in red/processed meat are associated with better treatment outcomes.

Additional hypotheses are that 1) low intake of red/processed meat and 2) high intake of dietary fibers, are independently associated with better treatment outcomes and 3) a potential interaction between them may further improve treatment outcomes.

## 2.5. Objective

To examine if treatment outcomes in patients with CID vary with dietary differences.

Primary objective: To compare the differences in clinical response following high intake of fiber *AND* low intake of red/processed meat, relative to a low intake of fiber *AND* high intake of red/processed meat, on the proportion of individuals achieving a clinical response (defined individually according to their specific condition) after a period of 14-16 weeks, in patients with CID.

Key secondary objectives: To compare the differences in clinical response following high intake of fiber *AND* low intake of red/processed meat, relative to a low intake of fiber *AND* high intake of red/processed meat, on changes in measures of health-related quality of life and disability (SF-12 physical component summary [PCS] and mental component summary [MCS], and the short health scale components), C-reactive protein (CRP), physician global assessment, and patient global assessment from baseline to week 14-16, as well as the proportion of individuals continuing biologic therapy at week 14-16, in patients with CID.

Exploratory secondary objectives: (*i*) To compare the effect of high intake of fiber relative to a low intake of fiber while adjusting for meat intake, on the proportion of individuals achieving a clinical response (defined individually according to their specific condition) after a period of 14-16 weeks, in patients with CID; (*ii*) To compare the effect of low intake of red/processed meat, relative to a high intake of red/processed meat while adjusting for fiber intake, on the proportion of individuals achieving a clinical response (defined individually according to their specific condition) after a period of 14-16 weeks, in patients with CID.

# 3. Study methods

## 3.1. Study design

BELIEVE is an observational multi-center cohort study with prospective enrollment of CD, UC, RA, axSpA, PsA and PsO patients initiating treatment with a biologic agent (or switching to another). Treatment with biologics was assigned to the patients independent of BELIEVE and their habitual diet. When a patient was found to be a candidate for initiation of biologic treatment or switching to another biologic agent, they were invited to participate in the study. Patients were examined at two time points; at baseline and 14-16 weeks after treatment initiation, according to Danish clinical standards. The examination program included questionnaires (including a Food Frequency Questionnaire at baseline), clinical assessment and sampling of blood, urine, feces and intestinal biopsies (the latter only for UC and CD). The inclusion period was between 1st of September 2017 and 31st of March 2020 with follow-up until end of July 2020. The following centers in Denmark participated in inclusion of patients to BELIEVE: Department of Hepatology and Gastroenterology, Aarhus University Hospital; Diagnostic Centre, Silkeborg Regional Hospital; Department of Gastroenterology, Herlev and Gentofte Hospital; Medical Department, Hospital Sønderjylland; Department of Gastroenterology, Hospital of South West Jutland; Department of Medical Gastroenterology, Department of Rheumatology and Department of Dermatology and Allergy Centre, Odense University Hospital.

## 3.2. Sample size and power considerations

Deciding on sample size is a well-known difficulty with exploratory prognostic factor research studies. To obtain an adequate number of outcome events, we applied 'the rule of thumb', whereby 10 outcomes are needed for

each independent variable. We planned to enroll 320 patients in total with the anticipation that 50% of these would experience a clinical response within the 14–16 weeks period after initiation of biological therapy. With this in mind and anticipating that we would observe at least 160 events (i.e., clinical response among the 320 patients), the study would be sufficiently powered to explore the impact of as many as 16 independent variables including condition and clinical center. Since using the 'rule of thumb' method to justify sample size is a debated practice, we went one step further and estimated the statistical power to detect differences between two dietary groups. For the contrast between groups and for a comparison of two independent binomial proportions (those with high-fiber, low-meat intake vs. other) using Pearson's $\chi^2$ statistic, with a $\chi^2$ approximation, with a two-sided significance level of 0.05 ($P < 0.05$), a total sample size of 318—assuming an 'allocation ratio' of 1 to 2 (one-third)—has an approximate power of 0.924 (i.e., >90% statistical power) if the anticipated proportions responding to therapy are 60% and 40%, respectively. However, due to pre-specified time constraints, inclusion to BELIEVE was terminated on 03-30-2020 before reaching the planned sample size of 320 patients in total.

## 3.3. Statistical interim analyses and stopping guidelines

No statistical interim analyses were planned or executed.

## 3.4. Timing of final analysis

The final analysis will be conducted after completion of the 14-16 week follow-up visit of the last patient included in the study (ultimo July 2020) and after completion of the SAP, approved and revised by the research team and collaborators. The present SAP pertain to the primary publication and analysis from the BELIEVE cohort study. Biological samples collected in BELIEVE (beside C-reactive protein [CRP]) are part of more exploratory objectives and the results will be presented in separate coming peer reviewed papers.

## 3.5. Timing of outcome assessments

As described in the pre-specified protocol [2], patients are examined at two time points; at baseline and 14-16 weeks after treatment initiation, where primary endpoints are evaluated.

Taking the COVID-19 pandemic into account, we have decided to allow a window of +/- 4 weeks for outcome assessment in order to prevent missing outcomes.

# 4. Statistical principles

## 4.1. Statistical significance and confidence intervals

All P values and 95% confidence intervals will be two sided. We will not apply explicit adjustments for multiplicity, rather we will interpret the analysis of the key secondary outcomes according to the Hochberg sequential procedure [21]: The analyses of the key secondary outcomes will be performed, and the correspondent P values will be ordered from largest to smallest in a list. If the largest P value is less than the significance level of 0.05, then all the tests are considered significant. In contrast, if the largest P value fails to show statistically significance, then progressively more stringent P values are applied as a significance level (the second largest P values: 0.05/2, the third largest: 0.05/3 and so on). When a test succeeds to show a statistically significant difference, then the remaining tests downstream from this are considered significant. Thus, comparisons continue until a test shows a statistical difference or until all comparisons are made. The key secondary statistical tests will be reported with P values for hypothesis tests and claims of potential statistical significance. Further, due to potential issues of multiplicity as multiple tests will be performed, we will interpret 'statistically significant' findings in the context of whether the 95% confidence interval (CI) excludes outcomes that could be perceived as clinically important.

## 4.2. Protocol deviations

### *Eligibility criteria*

**Protocol**: Eligible patients are patients diagnosed with a CID, including CD, UC, RA, axSpA, PsA, PsO, Hidradenitis Suppurativa (HS) and non-infectious Uveitis (niU) that are initiating biological therapy targeting TNF, who are naïve to biologics.

**SAP modifications:**

1) There were no patients with Hidradenitis Suppurativa and Non-infectious Uveitis included in the study.
2) Patients initiating all kinds of biological therapy were enrolled instead of only TNFi therapy*.
3) In addition, patients that previously have received biologic treatment were also made eligible*.

**We will apply these new strata in subsequent sensitivity analyses*

**Rationale:**

1) The Ophthalmology Department, who was expected to enroll patients with non-infectious uveitis did not engage in BELIEVE after all. In the period where The Department of Dermatology and Allergy Centre, OUH were engaged in enrollment of patients, there were too few patients with Hidradenitis Suppurativa initiating biological therapy.

2+3) We decided to broaden the inclusion criteria to increase the study population.

### *Enrollment period*

**Protocol:** Participant enrollment was expected to run from 1$^{st}$ of April 2017 to 31$^{st}$ of March 2019 or until a minimum of 100 patients with inflammatory bowel disease (CD and UC), 100 patients with RA, and 120 patients with axSpA, PsA, PsO, HS and niU were achieved.

**SAP:** The enrollment period was between 1$^{st}$ of September 2017 and 31$^{st}$ of March 2020.

**Rationale:** We extended the enrollment period with a year to increase the study population but did however not succeed to reach the planned sample size.

### *Primary endpoint*

**Protocol:** The disease specific primary endpoint for UC is a Mayo Clinic Index of 2 or less (with no individual subscore >1).

**SAP:** In cases, where a full Mayo Clinic Index is missing, we will accept a Partial Mayo Clinic Index, which is the Mayo Clinic Index without the endoscopic subscore. However, in these cases, the primary endpoint will be a Partial Mayo Clinic Index of 1 or less [22, 23].

**Rationale:** Some IBD patients refrained from repeated colonoscopies, hence hindering calculation of a full mayo score for UC patients.

*Timing of outcome assessment*

**Protocol:** Patients were examined at two time points; at baseline and 14-16 weeks after treatment initiation, where primary endpoints are evaluated.

**SAP:** Outcome assessment was scheduled to be 14-16 weeks after treatment initiation but a window of +/- 4 weeks are allowed.

**Rationale:** Taking the COVID-19 pandemic into account, we have decided to allow a window of +/- 4 weeks for outcome assessment at the follow-up visit in order to prevent a large number of missing outcomes.

## 4.3. Analysis populations

The primary analysis will be conducted according to the Intention-to-Treat (ITT) principle [24]. We define the ITT population as all CID participants who have signed informed consent and answered the FFQ (which will define the group allocation [the proxy for 'randomization'] of the participants in analogy to randomized trials) regardless of their adherence otherwise to the study protocol. A *per-protocol* population will be defined as those who adhered to the biological treatment during the observation period and have complete data used for the analyses at the end of the study, and no major protocol violations.

# 5. Study population

## 5.1. Screening data

The total number of patients initiating biological therapy screened for eligibility was not registered. An estimate will be calculated based on the usual number of patients initiating biological therapy per month at each clinical center multiplied with the number of months the center has participated in patient enrolment.

## 5.2. Eligibility

### 5.2.1. Inclusion criteria

- Patients with a verified diagnosis of CD, UC, RA, axSpA, PsA, PsO, HS or niU
- Initiation of biological therapy

### 5.2.2. Exclusion criteria

- Age <18 years
- Unable to read and understand Danish
- Mentally unable to answer the questionnaire

## 5.2. Recruitment

A flow chart adapted from http://www.consort-statement.org/ will be used to visualize the flow of participants; showing the number of people screened, reasons for ineligibility, the number of participants who consented and the number analyzed (summarized by type of exposure, i.e., dietary profile) (fig. 1).



CONFIDENTIAL

## 5.3. Adherence and reasons for withdrawal

The number of patients adhering to the protocol, i.e., those who continued the biological treatment from baseline to the follow-up visit, will be accounted for in the flowchart. Likewise, the flowchart will also account for the number of patients deviating from the protocol, i.e., they did not initiate biologic treatment after all, they ended treatment due to either adverse events or lack of efficacy, or they withdrew their consent (fig. 1).

## 5.4. Baseline patient characteristics

Baseline characteristics will be summarized by dietary profile and are displayed in table 1 (manuscript outline). The information is extracted from the baseline clinical assessment together with the baseline questionnaire which was sent electronically to the participants prior to treatment initiation. Data will be presented as means with standard deviations (SD) when normally distributed or as median with interquartile range in case of skewed data. Dichotomous and categorical variables will be presented in proportions.

The table will include information on age, sex, anthropometrics (height, weight and BMI), smoking status, diagnosis, disease duration, medication (current medication and number of previous biological medications used), patient reported outcome measures (short health scale, SF-12 physical component summary [PCS], SF-12 mental component summary [MCS]), nutrition (intake of energy, fiber and red and processed meat) as well as physician global assessment and CRP.

# 6. Analysis

## 6.1. Outcome definitions, measurement and calculation

### 6.1.1. Primary outcomes

The primary endpoint [2] is the proportion of patients with a clinical response to therapy 14-16 weeks after initiation of treatment with a biological agent. Thus, the primary outcome will be a composite depending on the disease specific definitions of clinical response, defined below:

- CD/Crohn's disease: clinical remission, defined as Harvey-Bradshaw Index (HBI) of 4 or less [25].
- UC/Ulcerative colitis: clinical remission, defined as Mayo Clinic Score of 2 or less (with no individual subscore of >1) [26-28].
- RA/Rheumatoid arthritis: clinical response, defined as at least a 20% improvement according to the criteria of the American College of Rheumatology (ACR20) [29, 30].
- AxSpA/Axial spondyloarthritis: clinical response, defined as at least a 20% improvement according to the Assessment of Spondyloarthritis International Society (ASAS20) [31, 32].
- PsA/Psoriatic arthritis: clinical response, defined as at least a 20% improvement according to the criteria of ACR20 [33].
- PsO/Psoriasis: clinical response, defined as at least a 75% improvement in the Psoriasis Area and Severity Index (PASI 75) [34].

For the measurement and calculation of HBI, Mayo Clinic Score and PASI see table A. ACR20 and ASAS20 are composite outcome measures defined below with composites further explained in table A.

ACR20 is a composite measure developed by the American College of Rheumatology for assessment of improvement of RA patients in clinical trials [30] but also used for assessment of PsA [33]. ACR20 is defined as ≥ 20% improvement in tender and swollen joint counts and ≥ 20% improvement in at least three out of the following five measures: 1) pain, 2) patient global assessment, 3) physician global assessment, 4) self-assessed

physical disability and 5) CRP or erythrocyte sedimentation rate. Information for calculation of ACR20 will be extracted from the Simplified Disease Activity Index (SDAI; patients and physicians global assessment and tender and swollen joint count) and the Health Assessment Questionnaire Disability Index (HAQ-DI; disability, see table A), while pain is a separate question in the self-administered questionnaire ("how much pain due to your arthritis do you suffer from, the past two days?") on a VAS 0-100 mm.

The ASAS20 improvement criteria are developed by the Assessment of SpondyloArthritis international Society and includes four domains [32]: 1) Patient Global Assessment, 2) Pain, 3) Function and 4) Inflammation. In order to meet the ASAS20 response, at least three of the four domains should improve by ≥ 20% and an absolute improvement of ≥ 10 units (on a 0-100 scale). In the remaining domain, there should be no worsening of ≥ 20% and ≥ 10 units (on a 0-100 scale). Information for calculation of ASAS20 is extracted from the Bath Ankylosing Spondylitis Disease Activity Index (BASDAI; pain and inflammation) and the Bath Ankylosing Spondylitis Functional Index (BASFI; function, see table A). Patient Global Assessment is extracted from a separate question in the self-administered questionnaire ("how much does your arthritis as a whole affect your life at present?") on a VAS 0-100 mm.

### 6.1.2. Key secondary outcome measures

Predefined key secondary outcomes include changes in generic outcomes from baseline to follow-up (14-16 weeks after treatment initiation) consisting of both clinical and patient reported outcomes (PRO):

- Health-related quality of life (HRQoL): the physical component summary from the Short Form Health Survey (SF-12 PCS)
- HRQoL: the mental component summary measure from the SF-12 (SF-12 MCS)
- HRQoL: symptom burden (from the short health scale [SHS])
- HRQoL (Disability): functional status (SHS)
- HRQoL: disease-related burden (SHS)
- HRQoL: general well-being (SHS)
- Acute phase reactant (CRP)
- Physician global assessment (0-100 mm VAS)
- Patient global assessment (0-100 mm VAS)

In addition, however not specifically defined in the published protocol:

- The proportion of patients continuing the biologic treatment (yes/no) at week 14-16.

The 12-item SF-12 is a shorter form of the Short Form 36 (SF-36) which is widely used for measuring HRQoL and has a range of 0-100 (higher values indicate better health), mean of 50 and a standard deviation of 10. The SF-12 questionnaire comprises questions about physical and social functioning over the past 4 weeks. From the SF-12, two summary measures can be estimated; PCS and MCS [35].

The Short Health Scale (SHS) is a four-item questionnaire assessing the patient's subjective experience of how their disease influences four health dimensions: 1) symptom burden (severity of symptoms, 0 = none, 100 = worst thinkable), 2) functional status (impact on daily activities, 0 = none, 100 = severely impaired function) , 3) disease-related burden (concern over illness, 0 = none, 100 = constantly) and 4) general well-being (0 = good, 100 = terrible) [36]. The four questions are graded from 0-100 and presented individually, where higher

scores indicate negative experience. The SHS has been validated as a measure for HRQoL for use in UC [36] and CD [37].

CRP: Blood samples as specified in the protocol were collected by a trained laboratory technician and handled according to set procedures. Results from analysis of blood samples will be limited to CRP (mg/L) in this study.

Physician global assessment and patient global assessment* is assessed on 100 mm VAS.

* Patient global assessment is not collected for PsO, CD and UC.

### 6.1.3. Other secondary outcomes

Other secondary outcomes are non-generic outcomes specific to a single or two of the CIDs and are listed in the table below (Table A). Unless otherwise specified, the outcomes are changes from baseline to follow-up. Some outcomes are a composite of other outcomes and are grouped together.

**Table A.** Other secondary outcomes with details on how to calculate and measure them.

| Outcome | Measurement and calculation | CID |
|---|---|---|
| HBI score | HBI are calculated based on five items. The patient is asked to score item 1-3 based on the previous day [27]: A) General well-being on a scale from 0 to 4 (0=very well, 4=terrible), B) Number of liquid stools per day (the number is included in the final score), C) Abdominal pain on a scale from 0 to 3 (0=none, 1=mild, 2=moderate, 3=severe), D) Abdominal mass (clinical assessment) on a scale from 0 to 3 (0=none, 1=dubious, 2=definite, 3=definite and tender), E) Extra-intestinal complications: arthralgia, uveitis, erythema nodosum, aphthous ulcers, pyoderma gangrenosum, anal fissure, new fistula, abscess (each selected item is awarded 1 point in the final score). The sum of the five items is the score. | CD |
| No. of draining fistulas | Clinical examination. | CD |
| Mayo Clinic Score<br><br>- "normal mucosal appearance" (findings on endoscopy) (proportion)<br><br>- Mayo Clinic response (proportion)<br>-Partial Mayo Clinic Score | A scoring system for assessment of UC activity. Calculation of the score is based on 4 items [28]: A) Stool frequency per day (0= normal no. of stools for this patient, 1= 1-2 stools more than normal, 2= 3-4 stools more than normal, 3= 5 or more stools more than normal), B) Rectal bleeding (0= no blood seen, 1= Streaks of blood with stool less than half the time, 2= obvious blood with stool most of the time, 3= blood alone passes), C) Findings on endoscopy (0= normal or inactive disease, 1=mild disease (erythema, decreased vascular pattern, mild friability), 2= moderate disease (marked erythema, absent vascular pattern, friability, erosions), 3= severe disease (spontaneous bleeding, ulcers)), and D) Physician's global assessment (0= normal, 1= mild disease, 2= moderate disease, 3= severe disease). We have decided that endoscopic subscores older than 3 months prior to treatment initiation are excluded.<br>**Response:** ≥3 point decrease in Mayo Clinic Score [26] (partial Mayo score: ≥2 point decrease [23]).<br><br>The Mayo Clinic Score when omitting the endoscopic subscore (C). | UC |
| STRIDE remission: Selecting Therapeutic Targets in Inflammatory Bowel Disease (proportion) | The STRIDE criteria for remission were developed by a group of IBD experts to achieve international consensus on appropriate evidence-based treatment targets [38]. The agreed upon target for CD is clinical/PRO remission (defined as resolution of abdominal pain and diarrhea/altered bowel habit, addressed in the HBI) and endoscopic remission (defined as resolution of ulceration at ileocolonoscopy) or resolution of findings of inflammation on cross-sectional imaging.<br>The target for UC is clinical/PRO remission (defined as resolution of rectal bleeding and diarrhea/altered bowel habit, addressed in the Mayo Clinic Score) and endoscopic remission (defined as Mayo endoscopic sub-score of 0-1) [38]. | CD<br>UC |
| Cortico-steroid free remission (proportion) | No. of patients reaching either HBI ≤ 4 or Mayo Clinic score ≤ 2 without concomitant use of steroids. | CD<br>UC |
| Concomitant medication | Change in number from baseline to follow up. | CD<br>UC |
| SCCAI:<br>Simple Clinical Colitis Activity Index | SCCAI is a tool developed to measure activity of UC [39]. It consists of six items: A) Bowel frequency (0-3= 0, 4-6= 1, 7-9= 2, >9= 3), B) Bowel frequency at night (0=0, 1-3= 1, 4-6= 2), C) Urgency of defecation (none= 0, hurry= 1, immediately= 2, incontinence= 3), D) Blood in stool (none= 0, trace= 1, occasionally frank= 2, usually frank= 3), E) General well-being (very well= 0, slightly below par= 1, poor= 2, very poor= 3, terrible= 4), Extra-colonic manifestations (Uveitis, pyoderma gangrenosum, erythema nodusum, arthropathy= 1 per manifestation). The sum of the six items is the total score and ranges from 0-19. | UC |
| HAQ-DI:<br>Health Assessment Questionnaire Disability Index<br><br>(Composite in | HAQ-DI is a validated PRO instrument for assessing disability in several rheumatic conditions [40]. The questionnaire includes items that assess fine movement of upper extremities, locomotor activities of lower extremities and activities involving both upper and lower extremities during the past week. Furthermore, it also includes items to assess the use of aids and devices or the assistance of another person. Each item is scored from 0 to 3 (0= without any difficulty, 1= with some difficulty, 2= with much | RA<br>PsA |

| | | |
|---|---|---|
| ACR20) | difficulty, 3= unable to do). The items in the questionnaire are divided into 8 functional categories; dressing, rising, eating, walking, hygiene, reach, grip, and usual activities with at least two questions within each category. When calculating the HAQ-DI score, the highest score within a category determines the category score. However, the use of aids, devices or physical assistance increases a score of zero or one to two. The mean of the scores for the 8 categories are the total score (0-3.0). For further details, see reference [40]. | |
| (DAS)28-CRP: Disease Activity Score | DAS28-CRP is a composite measure of disease activity in RA ranging on a scale from 0 to 9.4 [41] [42]. It is calculated using 4 variables (swollen and tender joint counts (SJC and TJC, 0-28), CRP (mg/L) and patient global assessment (PGA, 0-100 mm VAS))[41]: $DAS28(CRP) = \sqrt{TJC} + 0.28 * \sqrt{SJC} + 0.36 * \ln(CRP + 1) + 0.014 * PGA + 0.96$ | RA<br>PsA |
| SDAI:<br>Simplified Disease Activity Index<br><br>- Tender and Swollen joints<br>-Patient's Global Assessment | SDAI is also a composite measure for monitoring disease activity in RA. The index includes five clinical measures: swollen and tender joint count (0-28), patient global assessment of disease activity (10 cm VAS), provider global assessment of disease activity (10 cm VAS), and CRP (mg/dl). The sum of the five items is the SDAI score [42].<br>Tender and swollen joint counts + Patient's and Physician's Global Assessment are composites in ACR20. | RA<br>PsA |
| BASMI:<br>Bath Ankylosing Spondylitis Metrology Index | BASMI is a clinical measure for spinal mobility. Five clinical measurements are included: 1) lumbar side flexion (cm), 2) tragus to wall distance (cm), 3) lumbar flexion (cm, modified Scober), 4) intermalleolar distance (cm), and 5) cervical rotation (°) [32]. In Believe, a 3-point answer scale (0-2) is used (BASMI 3) based on specific categories corresponding to mild, moderate and severe disease for each of the five measurements. The sum of the five assessments is the BASMI 3 score [32]. | ax-SpA |
| BASFI:<br>Bath Ankylosing Spondylitis Functional Index | BASFI is a self-assessment instrument designed to define and monitor functional ability in patients with Ankylosing Spondylitis (AS). The BASFI comprises 10 questions on which respondents rate the degree of difficulty they have performing different tasks using VAS from 0 (easy) to 100 mm (impossible). The mean of the 10 responses is the BASFI score [43]. BASFi is a composite in the ASAS20 (function). | ax-SpA |
| BASDAI:<br>Bath Ankylosing Spondylitis Disease Activity Index<br><br>- Total score for back pain | BASDAI is also a self-assessment instrument used for evaluation of disease activity in ankylosing spondylitis. It is composed of six questions addressing five symptoms on a VAS (0-100 mm); severity of fatigue, spinal and peripheral joint pain, localized tenderness and morning stiffness (both duration and severity). The mean of the score for the five symptoms (i.e. the average of the two scores relating to morning stiffness is taken) is the BASDAI score [44].<br>ASAS20: inflammation; mean of BASDAI questions 5 and 6 regarding morning stiffness, pain; spinal joint pain (question 2) [32]. | ax-SpA |
| Psoriatic arthritis pain | A separate question in the self-administered questionnaire ("how much pain due to your arthritis do you suffer from, the past two days?") on a VAS 0-100 mm. | PsA<br>PsO |
| PASI score | PASI is a standard scoring tool used to measure the severity and extent of psoriasis ranging from 0 to 72. The four main body areas (the head: 10% of total body, the trunk: 20%, the upper extremities: 30% and the lower extremities: 40%) are each evaluated for area of psoriatic involvement with a numerical value from 0-6 corresponding to percentages of involvement in that area [45]. Next, the severity of the psoriatic lesions is evaluated for each body on three factors (erythema, infiltration and desquamation) on a scale from 0-4. For calculation of the PASI [45], for each body area, the sum of severity for these three factors are multiplied with the numerical value of involvement and the percentage of the total body. The sum of the values for the four body areas are the PASI score. | Pso<br>PsA |
| DLQI:<br>Dermatology Life Quality Index | DLQI is the most frequently used dermatology-specific measure of life quality in clinical trials of skin diseases [46]. It is composed of ten questions concerning the impact of the respondents' skin on different aspects of quality of life over the past week. Each item in the DLQI is scored on a 4-point scale (not relevant/not at all= 0, a little=1, a lot= 2, very much= 3), and the sum of the ten items is the total score (0-30) [46]. | PsO |

### 6.1.4. Exposure and confounding variables

***Exposure variable***

We will stratify the patients in BELIEVE into two groups (exposed versus unexposed) based on the ratio of fiber to meat intake (FtM [grams per day]):

$$\text{FtM} = \frac{Fibers}{Red\ and\ processed\ meat},$$

which will be calculated from the food frequency questionnaire (FFQ) administered to the patients at baseline. From this ratio, exposure is defined as the upper 33.3% of the total study sample (called the high fiber low meat group, HFLM) leaving the lower 66.6% of the sample as those un-exposed (low fiber high meat, LFHM).

Secondary explorative analysis will include defining exposure as 1) the upper 33.3% (high fiber; HF) of the study sample with respect to intake of dietary fibers (versus the lower 66.6% [low fiber; LF], and 2) the lower 33.3% (low meat; LM) of the study sample with regard to red/processed meat intake (versus the upper 66.6% [high meat; HM]).

The FFQ used in the present study was developed and validated in relation to the 2007-2008 Danish Health Examination Survey [47]. It was internet-based and administered to the participants prior to initiation of biological therapy with questions covering the habitual dietary intake during the past month. The FFQ contained information from 267 different food groups. For each item, frequency of consumption was evaluated by eight categories ranging from "newer/seldom" to "twice or more per day". In order to quantify portion sizes for main meals and other main food items, a photographic food atlas consisting of different food and meal series was included at the end of the questionnaire. However, fixed portion sizes were used for some food items with more standardized portion sizes such as fruits. The actual weight in grams for each food item will be derived by multiplying the reported frequency of consumption with estimated portion sizes. Total energy intake and intake of different nutrients will be quantified using the Danish Food Composition Tables (National Food Institute, Technical University of Denmark, https://frida.fooddata.dk/

***Confounding variables***

Randomized trials can be expensive, resource intensive to perform, and in certain circumstances unethical to perform; i.e., it might be difficult to ask (and trust) a large group of patients suffering from chronic inflammatory diseases to change their diet significantly (LFHM → HFLM, and vice versa). For these reasons, the BELIEVE study group have to rely on observational data. The challenge with observational data is that group allocation are not applied randomly, likely leading to selection bias with some corresponding confounding variables. In well performed Randomized Controlled Trials there will be no selection bias when splitting participants up into the control (e.g., LFHM) and treatment (e.g., HFLM) groups; it accounts for all possible "confounding variables"[48].

Improved confounding variable balance between exposure (HFLM) and control (LFHM) groups can be achieved by adjusting observations from each group based on the propensity score, which in this case would be the probability (1/3) that a patient received the experimental intervention (HFLM) given the observed covariates. Propensity score analysis seeks to isolate the treatment as the only difference between our treatment and control groups. Thus, propensity score methods attempt to correct for the assignment mechanism by providing a "balancing variable that creates" control units similar to treatment units at baseline (i.e. $Y_{0|\text{HFLM}} \approx Y_{0|\text{LFHM}}$).

Confounding variables are independent variables other than the treatment variable (LFHM and HFLM, respectively) that are correlated to the outcome of the study. In order to identify possible confounders and develop propensity scores, we will use the following pragmatic definition (illustrated below) of what potentially makes a confounding variable (*C*):

- The Covariate (*C*) is an ancestor (cause) of the outcome (*Y*)
- The Covariate (*C*) probably cause the exposure (*X*; e.g., group)
- The Covariate (*C*) is not a descendant (effect) of the exposure (*X*) or outcome (*Y*)

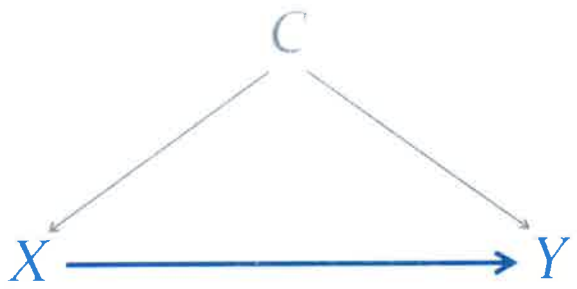


Variables we will test for possible confounding of the association between dietary profile and treatment response is sex, age, and smoking status [14, 15, 49, 50]. Other adjusting variables include CID type and clinical center.

## 6.2. Analysis methods

The baseline characteristics of the participants (table 1) will be summarized for each dietary exposure profile (HFLM and LFHM) using descriptive statistics. Data will be presented as either means and standard deviations (SDs), or medians and interquartile ranges (IQRs) for continuous outcomes, and numbers and percentages for binary outcomes.

The primary outcome is the proportion of patients with a clinical response to therapy 14-16 weeks after treatment initiation. Response is defined for each CID (see section 6.1.1.). The number and proportion of participants who achieved treatment response will be summarized based on dietary profile (fig. 2 and table 2). We will investigate the association between dietary profile at baseline (HFLM and LFHM) and treatment response (yes/no) on the ITT population using logistic regression models. A simple (unadjusted) logistic regression model, an adjusted model (adjusted for type of CID, clinical center, age, sex, smoking) and a propensity adjusted model (further adjusted for propensity score) will be presented in table 2. The Odds ratio (OR) of achieving clinical response at week 14-16 for CID patients in HFLM group (the upper 33.3 % of the study population based on the ratio of fiber to meat intake) versus the LFHM group (the lower 66.6 %) will be reported along with the 95 % confidence interval (CI) and two-sided p-value (fig. 2 and table 2). We will also perform similar analyses on the key secondary outcome measures (see 6.1.2.), corresponding to our pre-specified key secondary objectives.

Secondary explorative analyses will include repeating the logistic regression models for treatment outcomes using secondary predictors of interest; 1) the upper 33.3% (high fiber; HF) vs. the lower 66.6% (low fiber; LF) of the study sample with respect to intake of dietary fibers (adjusted for meat intake), and 2) the lower 33.3% (low meat; LM) vs. the upper 66.6% (high meat; HM) of the study sample with regard to red/processed meat intake (adjusted for fiber intake). In addition, a possible interaction between the lower 33.3% of the sample

with respect to red/processed meat intake and the upper 33.3% of the sample with regard to fiber intake will be examined using logistic regression with same covariates as stated above.

The result from the primary and key secondary outcomes will be presented in table 2, while other secondary outcomes will be presented in a separate table (Appendix table 1). Results from the secondary explorative analyses will be presented in table 3 and 4 in the Appendix.

## 6.3. Missing data

As previously stated (under section 4.3), the primary analyses will be based on the ITT population. Accordingly, participants will be followed up, assessed and analyzed as members of their initial dietary group, irrespective of their adherence to the planned course of treatment with biological agents and potential personal changes in dietary habits (i.e., independent of withdrawals and cross-over phenomena). Every effort will be made to minimize missing data (i.e. both baseline covariates [*X*'s] and outcome data [*Y*'s]). Loss to follow-up and missing data for various reasons is however difficult to avoid in pragmatic, real-world observational studies, from clinical practice.

We will apply the analysis framework suggested by White et al (2011) in which missing data related to the ITT approach depend on making plausible assumptions about the missingness of the data and including all participants in subsequent sensitivity analyses [24]:

**#1. *Attempt to follow up all participants who signed informed consent*,** even if they withdrew from allocated treatment or significantly modified their dietary habits (i.e., we contact all individuals unless they explicitly stated that they had withdrawn their consent).

**#2. *Perform a main analysis of all observed data that are valid under a plausible assumption.*** For our primary analyses we will assume that data is 'Missing At Random' (MAR). The MAR assumption requires that, conditional on the observed data for the case, $Y_{obs}$, the probability that a value is missing does not depend on the true values of the missing items, $Y_{mis}$. Multiple imputation (MI) will be used to account for participants from whom we have an answered FFQ (i.e. the ITT population) but potentially with missing outcome information at follow-up.

**#3. *Perform sensitivity analyses.*** In order to explore the effect of departures from the assumption made in the main analysis (#2) other analysis techniques will be applied; these models will potentially be informative even if data are '*Missing Not At Random*' [MNAR].

***Use and reporting of analyses using multiple imputation.*** When we assume data are missing at random (MAR), then unbiased and statistically more powerful analyses is possible by building a more general model based on multiple imputation. Multiple imputation (MI) is a general approach to the problem of missing data that allow for the uncertainty about the missing data by creating several different plausible imputed data sets and appropriately combining results obtained from each of them.

The *MI first stage* will be to create multiple (i.e. at least 5) copies of the dataset, with the missing values replaced by imputed values. These will be sampled from their predictive distribution based on the observed data.

The *MI second stage* will be to use standard statistical methods to fit the logistic regression model of interest to each of the imputed datasets; variables included in the imputation models will be listed. At least five imputed datasets will be created. Estimated associations (i.e. ln[$OR_i$]) in each of the imputed datasets will differ

because of the variation introduced in the imputation of the missing values, and they are only useful when averaged together to give overall estimated associations. Standard errors will be calculated using Rubin's rules [51] which take account of the variability in results between the imputed datasets, reflecting the uncertainty associated with the missing values. Valid inferences are obtained because we will be averaging over the distribution of the missing data given the observed data.

We will report the number of missing values for each variable of interest, and the number of cases with complete data for each important component of the analysis. We will provide reasons for missing values if possible and indicate how many individuals were excluded because of missing data when reporting the flow of participants through the study (Figure 1). We will provide explicit reasons for missing data for the primary outcome measure in terms of other variables and explore whether there are differences between individuals with complete and incomplete primary outcome data, by performing logistic regression analyses with "missingness" being the dependent variable ($Y_{\text{Mis}}$ *vs.* $Y_{\text{Obs}}$) and various baseline characteristics (covariates) as independent variables.

The selection of the variables to be used in the imputation of missing data will follow the following three basic rules, that will enable us to perform multiple imputation and analysis that is statistically robust:

1. Include all the key analysis variables, regardless of whether they have missing data;

2. Include other variables that are correlated or associated with the primary outcome we intend to analyze (regardless of whether these variables will ultimately be included in our analysis);

3. Include variables that predict item missing data on the analysis variables (again regardless of whether these variables will ultimately enter our analysis).

We will report the details of the software used and of key settings for the imputation modeling; incl. the number of imputed datasets that were created. We will use mainly default options but do specify a specific SEED value (e.g. the specific date of the analysis); this seed will be used to initiate the generation of random numbers that are used in the imputation process. The specification of a fixed SEED value enables us to exactly replicate the random number generation sequence and reproduce the results of a multiple imputation analysis run. We will use the following default settings: M=5 repetitions and 20 burn-in iterations.

Since our analytic objective is to estimate the Odds Ratios (from logistic regression) of clinical response depending on the dietary group (and other covariates simultaneously), we will use logistic regression with a "BY statement" to execute five separate logistic regressions, one for each imputation repetition data set contained in the corresponding "MI data set".

The *MI third and final step* in our analysis plan is to combine these five sets of regression estimates to produce the final MI estimates of the logistic regression model parameters, their standard errors, and 95% CIs for the true parameter values. As a consequence, the parameter estimates from the five individual logistic regressions are averaged and the associated standard errors account for the imputation variability.

## 6.4. Sensitivity analyses

Where complete cases and multiple imputation analyses give different results, the analyst should attempt to understand why, and this should be reported in publications [52]. Robustness is a concept that refers to the sensitivity of the overall conclusions to various limitations of the data, assumptions, and analytic approaches to data analysis. Robustness implies that the treatment effect and primary conclusions of the trial are not

substantially affected when analyses are carried out based on alternative assumptions or analytic approaches. For the purpose of sensitivity, multiple sensitivity analyses will be performed to assess the robustness of the primary analyses. For instance, a simplistic 'single step, null responder imputation' would represent a conservative base case and is potentially valid even if data is 'missing not at random' as it assumes and implies that patients have not improved or worsened after entering the study. When there are missing outcome data for our dichotomous outcomes we will to explore "best" and "worst" case scenarios by replacing missing values with "good" outcomes in one group and "bad" outcomes in the other group. Sensitivity analyses will include analyses based on:

1) complete case analysis,
2) a 'non-responder-imputation',
3) the 'per protocol population',
4) the population meeting the unmodified eligibility criteria (exclusion of patients initiating treatment with biologics targeting other agents than TNF and patients previously treated with biologics).

Results of the sensitivity analyses are presented in the Appendix, table 2. When the different sensitivity analyses are in agreement, and the analyses on the sensitivity analyses and the main analysis lead to essentially the same conclusions, confidence in the trial results is increased.

## 6.5. Statistical software

Statistical programming will be done using the software STATA and R with transparent reporting of the source code used to analyze the data.

# 7. References


1. Gamble, C., et al., *Guidelines for the Content of Statistical Analysis Plans in Clinical Trials.* Jama, 2017. **318**(23): p. 2337-2343.
2. Christensen, R., et al., *Impact of red and processed meat and fibre intake on treatment outcomes among patients with chronic inflammatory diseases: protocol for a prospective cohort study of prognostic factors and personalised medicine.* BMJ Open, 2018. **8**(2): p. e018166.
3. Vandenbroucke, J.P., et al., *Strengthening the Reporting of Observational Studies in Epidemiology (STROBE): explanation and elaboration.* Ann Intern Med, 2007. **147**(8): p. W163-94.
4. Moons, K.G., et al., *Transparent Reporting of a multivariable prediction model for Individual Prognosis or Diagnosis (TRIPOD): explanation and elaboration.* Ann Intern Med, 2015. **162**(1): p. W1-73.
5. Baumgart, D.C. and S.R. Carding, *Inflammatory bowel disease: cause and immunobiology.* Lancet, 2007. **369**(9573): p. 1627-40.
6. Baumgart, D.C. and W.J. Sandborn, *Inflammatory bowel disease: clinical aspects and established and evolving therapies.* Lancet, 2007. **369**(9573): p. 1641-57.
7. Taurog, J.D., A. Chhabra, and R.A. Colbert, *Ankylosing Spondylitis and Axial Spondyloarthritis.* N Engl J Med, 2016. **374**(26): p. 2563-74.
8. Cross, M., et al., *The global burden of rheumatoid arthritis: estimates from the global burden of disease 2010 study.* Ann Rheum Dis, 2014. **73**(7): p. 1316-22.
9. Parisi, R., et al., *Global epidemiology of psoriasis: a systematic review of incidence and prevalence.* J Invest Dermatol, 2013. **133**(2): p. 377-85.
10. Kaplan, G.G., *The global burden of IBD: from 2015 to 2025.* Nat Rev Gastroenterol Hepatol, 2015. **12**(12): p. 720-7.
11. Nielsen, O.H. and M.A. Ainsworth, *Tumor necrosis factor inhibitors for inflammatory bowel disease.* N Engl J Med, 2013. **369**(8): p. 754-62.
12. Aletaha, D. and J.S. Smolen, *Diagnosis and Management of Rheumatoid Arthritis: A Review.* Jama, 2018. **320**(13): p. 1360-1372.
13. Digby-Bell, J.L., et al., *Interrogating host immunity to predict treatment response in inflammatory bowel disease.* Nat Rev Gastroenterol Hepatol, 2020. **17**(1): p. 9-20.
14. Furman, D., et al., *Chronic inflammation in the etiology of disease across the life span.* Nat Med, 2019. **25**(12): p. 1822-1832.
15. Ananthakrishnan, A.N., *Epidemiology and risk factors for IBD.* Nat Rev Gastroenterol Hepatol, 2015. **12**(4): p. 205-17.
16. Scott, D.L., F. Wolfe, and T.W. Huizinga, *Rheumatoid arthritis.* Lancet, 2010. **376**(9746): p. 1094-108.
17. Lahiri, M., et al., *Using lifestyle factors to identify individuals at higher risk of inflammatory polyarthritis (results from the European Prospective Investigation of Cancer-Norfolk and the Norfolk Arthritis Register--the EPIC-2-NOAR Study).* Ann Rheum Dis, 2014. **73**(1): p. 219-26.
18. Piovani, D., et al., *Environmental Risk Factors for Inflammatory Bowel Diseases: An Umbrella Review of Meta-analyses.* Gastroenterology, 2019. **157**(3): p. 647-659.e4.
19. Vedamurthy, A. and A.N. Ananthakrishnan, *Influence of Environmental Factors in the Development and Outcomes of Inflammatory Bowel Disease.* Gastroenterol Hepatol (N Y), 2019. **15**(2): p. 72-82.
20. Andersen, V., A.K. Hansen, and B.L. Heitmann, *Potential Impact of Diet on Treatment Effect from Anti-TNF Drugs in Inflammatory Bowel Disease.* Nutrients, 2017. **9**(3).
21. Cao, J. and S. Zhang, *Multiple comparison procedures.* Jama, 2014. **312**(5): p. 543-4.
22. Walsh, A.J., R.V. Bryant, and S.P. Travis, *Current best practice for disease activity assessment in IBD.* Nat Rev Gastroenterol Hepatol, 2016. **13**(10): p. 567-79.
23. Turner, D., et al., *A systematic prospective comparison of noninvasive disease activity indices in ulcerative colitis.* Clin Gastroenterol Hepatol, 2009. **7**(10): p. 1081-8.
24. White, I.R., et al., *Strategy for intention to treat analysis in randomised trials with missing outcome data.* Bmj, 2011. **342**: p. d40.
25. Khanna, R., et al., *Early combined immunosuppression for the management of Crohn's disease (REACT): a cluster randomised controlled trial.* Lancet, 2015. **386**(10006): p. 1825-34.

26. Vermeire, S., et al., *Etrolizumab as induction therapy for ulcerative colitis: a randomised, controlled, phase 2 trial.* Lancet, 2014. **384**(9940): p. 309-18.
27. Harvey, R.F. and J.M. Bradshaw, *A simple index of Crohn's-disease activity.* Lancet, 1980. **1**(8167): p. 514.
28. Schroeder, K.W., W.J. Tremaine, and D.M. Ilstrup, *Coated oral 5-aminosalicylic acid therapy for mildly to moderately active ulcerative colitis. A randomized study.* N Engl J Med, 1987. **317**(26): p. 1625-9.
29. Taylor, P.C., et al., *Baricitinib versus Placebo or Adalimumab in Rheumatoid Arthritis.* N Engl J Med, 2017. **376**(7): p. 652-662.
30. Felson, D.T., et al., *American College of Rheumatology. Preliminary definition of improvement in rheumatoid arthritis.* Arthritis Rheum, 1995. **38**(6): p. 727-35.
31. Baeten, D., et al., *Secukinumab, an Interleukin-17A Inhibitor, in Ankylosing Spondylitis.* N Engl J Med, 2015. **373**(26): p. 2534-48.
32. Sieper, J., et al., *The Assessment of SpondyloArthritis international Society (ASAS) handbook: a guide to assess spondyloarthritis.* Ann Rheum Dis, 2009. **68 Suppl 2**: p. ii1-44.
33. Mease, P.J., et al., *Secukinumab Inhibition of Interleukin-17A in Patients with Psoriatic Arthritis.* N Engl J Med, 2015. **373**(14): p. 1329-39.
34. Lebwohl, M., et al., *Phase 3 Studies Comparing Brodalumab with Ustekinumab in Psoriasis.* N Engl J Med, 2015. **373**(14): p. 1318-28.
35. Gandek, B., et al., *Cross-validation of item selection and scoring for the SF-12 Health Survey in nine countries: results from the IQOLA Project. International Quality of Life Assessment.* J Clin Epidemiol, 1998. **51**(11): p. 1171-8.
36. Hjortswang, H., et al., *The Short Health Scale: a valid measure of subjective health in ulcerative colitis.* Scand J Gastroenterol, 2006. **41**(10): p. 1196-203.
37. Stjernman, H., et al., *Short health scale: a valid, reliable, and responsive instrument for subjective health assessment in Crohn's disease.* Inflamm Bowel Dis, 2008. **14**(1): p. 47-52.
38. Peyrin-Biroulet, L., et al., *Selecting Therapeutic Targets in Inflammatory Bowel Disease (STRIDE): Determining Therapeutic Goals for Treat-to-Target.* Am J Gastroenterol, 2015. **110**(9): p. 1324-38.
39. Walmsley, R.S., et al., *A simple clinical colitis activity index.* Gut, 1998. **43**(1): p. 29-32.
40. Bruce, B. and J.F. Fries, *The Health Assessment Questionnaire (HAQ).* Clin Exp Rheumatol, 2005. **23**(5 Suppl 39): p. S14-8.
41. Madsen, O.R., *Agreement between the DAS28-CRP assessed with 3 and 4 variables in patients with rheumatoid arthritis treated with biological agents in the daily clinic.* J Rheumatol, 2013. **40**(4): p. 379-85.
42. Anderson, J.K., et al., *Measures of rheumatoid arthritis disease activity: Patient (PtGA) and Provider (PrGA) Global Assessment of Disease Activity, Disease Activity Score (DAS) and Disease Activity Score with 28-Joint Counts (DAS28), Simplified Disease Activity Index (SDAI), Clinical Disease Activity Index (CDAI), Patient Activity Score (PAS) and Patient Activity Score-II (PASII), Routine Assessment of Patient Index Data (RAPID), Rheumatoid Arthritis Disease Activity Index (RADAI) and Rheumatoid Arthritis Disease Activity Index-5 (RADAI-5), Chronic Arthritis Systemic Index (CASI), Patient-Based Disease Activity Score With ESR (PDAS1) and Patient-Based Disease Activity Score without ESR (PDAS2), and Mean Overall Index for Rheumatoid Arthritis (MOI-RA).* Arthritis Care Res (Hoboken), 2011. **63 Suppl 11**: p. S14-36.
43. Calin, A., et al., *A new approach to defining functional ability in ankylosing spondylitis: the development of the Bath Ankylosing Spondylitis Functional Index.* J Rheumatol, 1994. **21**(12): p. 2281-5.
44. Garrett, S., et al., *A new approach to defining disease status in ankylosing spondylitis: the Bath Ankylosing Spondylitis Disease Activity Index.* J Rheumatol, 1994. **21**(12): p. 2286-91.
45. Fredriksson, T. and U. Pettersson, *Severe psoriasis--oral therapy with a new retinoid.* Dermatologica, 1978. **157**(4): p. 238-44.
46. Basra, M.K., et al., *A review of the use of the dermatology life quality index as a criterion in clinical guidelines and health technology assessments in psoriasis and chronic hand eczema.* Dermatol Clin, 2012. **30**(2): p. 237-44, viii.
47. Eriksen, L., et al., *The Danish Health Examination Survey 2007-2008 (DANHES 2007-2008).* Scand J Public Health, 2011. **39**(2): p. 203-11.
48. Moher, D., et al., *CONSORT 2010 explanation and elaboration: updated guidelines for reporting parallel group randomised trials.* Bmj, 2010. **340**: p. c869.

49. Deane, K.D., et al., *Genetic and environmental risk factors for rheumatoid arthritis.* Best Pract Res Clin Rheumatol, 2017. **31**(1): p. 3-18.
50. Alkerwi, A., et al., *Smoking status is inversely associated with overall diet quality: Findings from the ORISCAV-LUX study.* Clin Nutr, 2017. **36**(5): p. 1275-1282.
51. Rubin, D.B., *Multiple Imputation for Nonresponse in Surveys*. Wiley Series in probability and mathematical statistics. Applied probability and statistics. 1987, New York, United States of America: Wiley. 253.
52. Sterne, J.A., et al., *Multiple imputation for missing data in epidemiological and clinical research: potential and pitfalls.* Bmj, 2009. **338**: p. b2393.

# 8. Manuscript outline

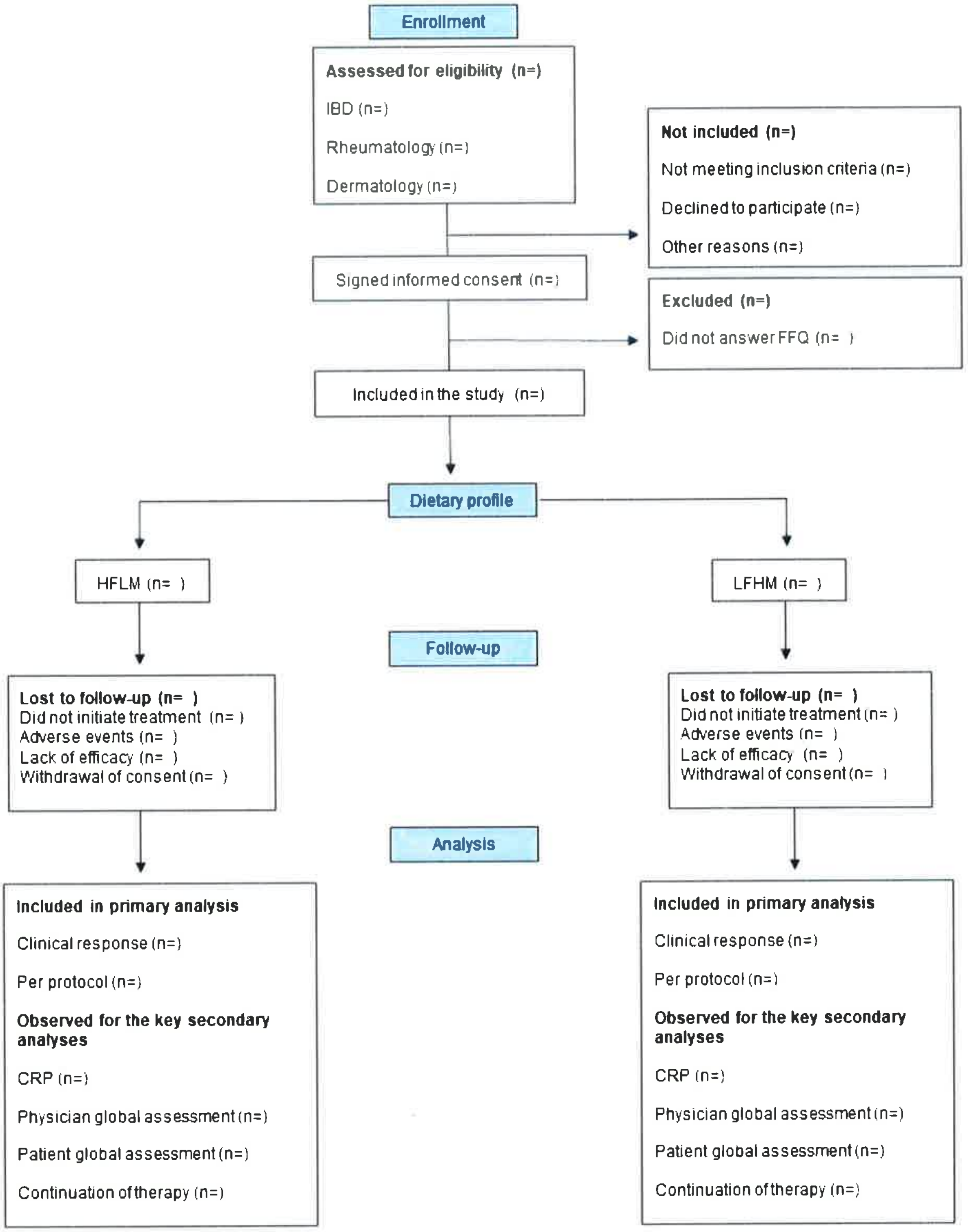


**Figure 1. Flow chart of the recruitment of participants.** IBD; Inflammatory bowel disease (Crohn's disease and Ulcerative colitis), FFQ; Food Frequency Questionnaire, HFLM; High fiber low meat (the exposed group: the upper 33.3 % of the study sample based on the ratio of fiber to meat intake), LFHM; low fiber high meat (the unexposed group; the lower 66.6 % of the study sample based on the ratio of fiber to meat intake), CRP; C-reactive protein.

**Table 1. Baseline characteristics of included participants.**

| Characteristic | HFLM (n=) | LFHM (n=) | *Difference between distributions | Total (n=) |
|---|---|---|---|---|
| Age, mean (SD), years | | | | |
| Female, n (%) | | | | |
| **Anthropometrics** | | | | |
| Height, mean (SD), cm | | | | |
| Weight, mean (SD), Kg | | | | |
| BMI (kg/m$^2$), mean (SD) | | | | |
| **Smoking status, n (%)** | | | | |
| Daily | | | | |
| Occasionally | | | | |
| Former | | | | |
| Non-smoker | | | | |
| **CID diagnose, n (%)** | | | | |
| Crohn's disease | | | | |
| Ulcerative collitis | | | | |
| Rheumatoid arthritis | | | | |
| Axial spondyloarthritis | | | | |
| Psoriatic arthritis | | | | |
| Psoriasis | | | | |
| **Disease duration, n (%), years:** | | | | |
| <1 | | | | |
| 1–4 | | | | |
| 5–9 | | | | |
| 10–19 | | | | |
| ≥20 | | | | |
| Disease duration (years), median (IQR) | | | | |
| **No of previous biological medications used (if any), n (%):** | | | | |
| 0 | | | | |
| 1 | | | | |
| 2 | | | | |
| ≥3 | | | | |
| **Medication, n (%):** | | | | |
| None | | | | |
| NSAID, daily use | | | | |
| Corticosteroids | | | | |
| Immunomodulators | | | | |
| 5-aminosalicyclic acid/sulfasalazine | | | | |
| Antibiotics | | | | |
| Hydroxychloroquin | | | | |
| Leflunomid | | | | |
| **PROs** | | | | |
| HRQoL | | | | |
| SF-12 PCS (0-100), median (IQR) | | | | |
| SF-12 MCS (0-100), median (IQR) | | | | |
| Symptom burden (0-100), median (IQR) | | | | |

| |
|---|
| Functional status (0-100), median (IQR) |
| Disease-related burden (0-100), median (IQR) |
| General well-being (0-100), median (IQR) |
| Patient global assessment (0-100), median (IQR) |
| **Nutrition** |
| Energy, mean (SD), kJ/d |
| Fibre, mean (SD), g/d |
| Red/processed meat, mean (SD), g/d |
| **Clinical examination** |
| Physician global assessment (0-100 mm VAS), median (IQR) |
| CRP, mean (SD), mg/L |
| *Will be estimated by comparing groups using the standardized mean difference (SMD); an SMD above 0.5 SD-units will be evaluated as a potential (data driven) confounding variable. HFLM; High fiber/low meat (exposed), LFHM; low fiber/High meat, CID; Chronic Inflammatory Disease, NSAID; Non-steroidal Anti-Inflammatory Drugs, PROs; patient reported outcomes, HRQoL; Health related quality of life, SF-12; 12-item Short Form survey, PCS; physical component summary, MCS; mental component summary, VAS; visual analog scale, CRP; C-reactive protein |

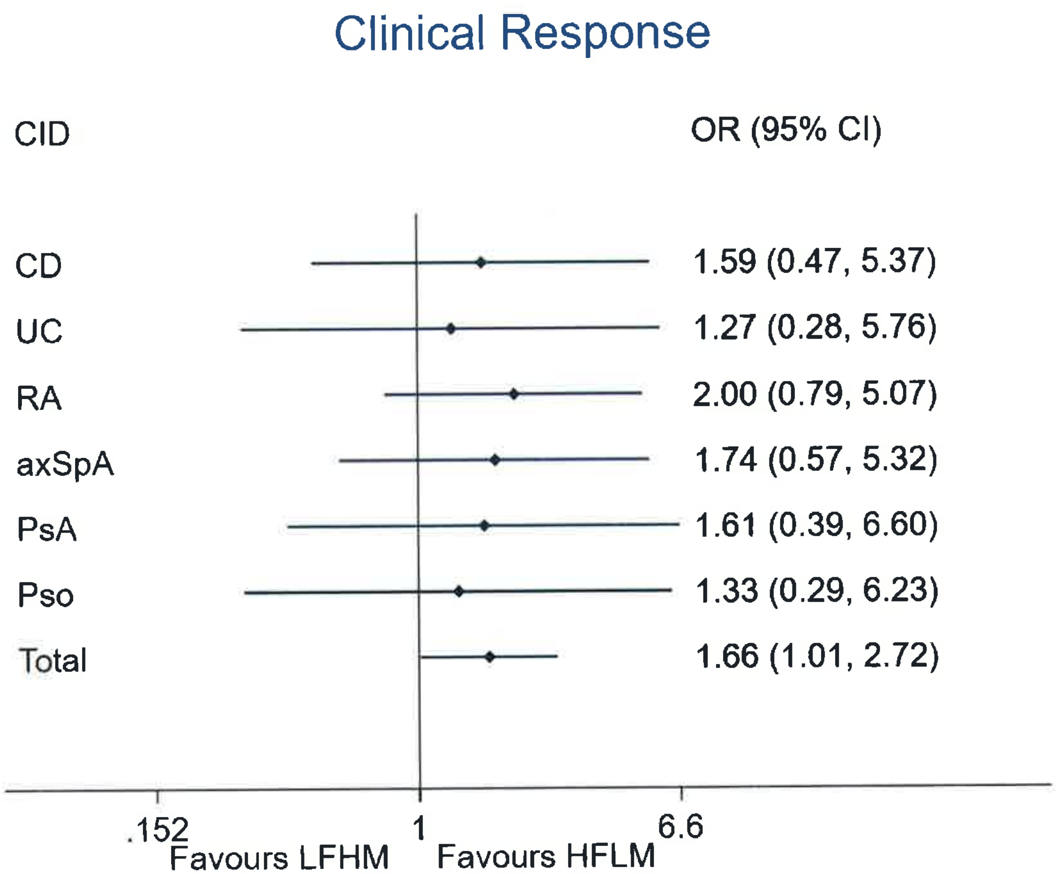


**Figure 2. Forest plot; Effect of dietary profile on treatment response. Data are simulated for the purpose of visualization.** CID; Chronic Inflammatory Disease, OR; Odds ratio, CI; Confidence interval, CD; Crohn's disease, UC; Ulcerative Collitis, RA; Rheumatoid Arthritis, axSpA; axial Spondyloarthritis, PsA; Psoriatic arthritis, Pso; Psoriasis, FFQ; Food Frequency Questionnaire, HFLM; High fiber low meat (the exposed group: the upper 33.3 % of the study sample based on the ratio of fiber to meat intake), LFHM; low fiber high meat (the unexposed group; the lower 66.6 % of the study sample based on the ratio of fiber to meat intake).



CONFIDENTIAL

Table 2. Comparison of high fiber and low meat (HFLM) versus low fiber and high meat (LFHM) with regard to clinical response and key secondary outcomes.

| | | | Unadjusted (crude) model[1] | | Adjusted model[2] | | Propensity adjusted model[3] | |
|---|---|---|---|---|---|---|---|---|
| **Outcome** | **HFLM** | **LFHM** | **Difference (95% CI)** | **P value*** | **Difference (95% CI)** | **P value*** | **Difference (95% CI)** | **P value*** |
| **Primary outcome (Composite outcome)** | | | | | | | | |
| Clinical response, n (%) | | | | | | | | |
| ***Sub-components*** | | | | | | | | |
| HBI≤4, n (%) | | | | n.a. | | n.a. | | n.a. |
| Mayo≤2, n (%) | | | | n.a. | | n.a. | | n.a. |
| ACR20 response, n (%) | | | | n.a. | | n.a. | | n.a. |
| ASAS20 response, n (%) | | | | n.a. | | n.a. | | n.a. |
| PASI75 response, n (%) | | | | n.a. | | n.a. | | n.a. |
| ***Key secondary outcomes:*** | | | | | | | | |
| *Health-related quality of life:* | | | | | | | | |
| ΔSF-12 PCS (0-100) | | | | | | | | |
| ΔSF-12 MCS (0-100) | | | | | | | | |
| ΔSymptom burden (0-100) | | | | | | | | |
| ΔFunctional status (0-100) | | | | | | | | |
| ΔDisease-related burden (0-100) | | | | | | | | |
| ΔGeneral well-being (0-100) | | | | | | | | |
| ΔCRP (mg/L) | | | | | | | | |
| ΔPhysicians global assessment (0-100 mm VAS) | | | | | | | | |
| ΔPatient global assessment (0-100 mm VAS) | | | | n.a. | | n.a. | | n.a. |
| Continuation of treatment, n (%) | | | | | | | | |
| ***Safety/Harms:*** | | | | | | | | |
| Withdrawals, n (%) | | | | n.a. | | n.a. | | n.a. |
| Withdrawals due to adverse events, n (%) | | | | n.a. | | n.a. | | n.a. |
| Serious adverse events (SAEs), n (%) | | | | n.a. | | n.a. | | n.a. |

*Key secondary outcomes are interpreted based on the Benjamini Hochberg approach. [1]Adjusted for CID type. [2]Adjusted for CID type, age, sex, clinical center and smoking. [3]Additionally adjusted for propensity score. HFLM; High fiber/low meat group, LFHM; Low fiber/high meat, CI; confidence interval, HBI; Harvey Bradshaw Index, Mayo; Mayo Clinic Score, ACR20; 20% improvement according to the criteria of the American College of Rheumatology, ASAS20; 20% improvement according to Assessment of Spondyloarthritis International Society, PASI75; 75% improvement in the Psoriasis Area and Severity Index, CRP; C-reactive protein, VAS; visual analog scale, SF-12; 12-item Short Form survey, PCS; physical component summary, MCS; mental component summary.

# Appendix

Appendix Table 1. Comparison of high fiber and low meat (HFLM) versus low fiber and high meat (LFHM) with regard to other secondary outcomes (i.e. disease specific outcomes)

| Outcome | N(HFLM) | HFLM | N(LFHM) | LFHM | Difference (95% CI) |
|---|---|---|---|---|---|
| ΔHBI, score | | | | | |
| ΔNo. of draining fistulas | | | | | |
| ΔMayo Clinic Score (0-12) | | | | | |
| Mayo Clinic Response, n (%) | | | | | |
| STRIDE remission, n (%) | | | | | |
| Cortico-seroid free remission, n (%) | | | | | |
| ΔConcomitant medication | | | | | |
| ΔSCCAI, score (0-19) | | | | | |
| ΔSDAI, score (0-86) | | | | | |
| ΔSwollen joint count (0-28) | | | | | |
| ΔTender joint count (0-28) | | | | | |
| ΔHAQ-DI, score (0-3) | | | | | |
| ΔDAS28-CRP, score (0-9.4) | | | | | |
| ΔBASMI (0-10) | | | | | |
| ΔBASFI (0-100) | | | | | |
| ΔBASDAI (0-100) | | | | | |
| ΔTotal score for back pain (0-100 mm VAS) | | | | | |
| ΔPASI score (0-72) | | | | | |
| Δ Psoriatic arthritis pain (100 mm VAS) | | | | | |
| ΔDLQI (0-30) | | | | | |

HFLM; high fiber low meat, LFHM; low fiber high meat, CI; confidence interval, HBI; Harvey Bradshaw Index, STRIDE; Selecting Therapeutic Targets in Inflammatory Bowel Disease, SCCAI; Simple Clinical Colitis Activity Index, SDAI; Simplified Disease Activity Index, HAQ-DI; Health Assessment Questionnaire Disability Index, DAS28-CRP; Disease Activity Score 28 - C-Reactive Protein, BASMI; Bath Ankylosing Spondylitis Metrology Index, BASFI; Bath Ankylosing Spondylitis Functional Index, BASDAI; Bath Ankylosing Spondylitis Disease Activity Index, PASI; Psoriasis Area and Activity index, DLQI; Dermatology Life Quality Index.



CONFIDENTIAL

Appendix Table 2. Sensitivity analyses of the comparison of HFLM versus LFHM.

| | | | Complete-case analysis | | Non-responder imputation analyses | | Per protocol population | | Unmodified eligibility criteria | |
|---|---|---|---|---|---|---|---|---|---|---|
| **Outcome** | **HFLM** | **LFHM** | **Difference (95% CI)** | **P value*** | **Difference (95% CI)** | **P value*** | **Difference (95% CI)** | **P value*** | **Difference (95% CI)** | **P value*** |
| **Primary outcome (Composite outcome)** | | | | | | | | | | |
| Clinical response, n (%) | | | | | | | | | | |
| ***Sub-components*** | | | | | | | | | | |
| HBI≤4, n (%) | | | | n.a. | | n.a. | | n.a. | | n.a. |
| Mayo≤2, n (%) | | | | n.a. | | n.a. | | n.a. | | n.a. |
| ACR20 response, n (%) | | | | n.a. | | n.a. | | n.a. | | n.a. |
| ASAS20 response, n (%) | | | | n.a. | | n.a. | | n.a. | | n.a. |
| PASI75 response, n (%) | | | | n.a. | | n.a. | | n.a. | | n.a. |
| ***Key secondary outcomes:*** | | | | | | | | | | |
| *Health-related quality of life:* | | | | | | | | | | |
| ΔSF-12 PCS (0-100) | | | | | | | | | | |
| ΔSF-12 MCS (0-100) | | | | | | | | | | |
| ΔSymptom burden (0-100) | | | | | | | | | | |
| ΔFunctional status (0-100) | | | | | | | | | | |
| ΔDisease-related burden (0-100) | | | | | | | | | | |
| ΔGeneral well-being (0-100) | | | | | | | | | | |
| ΔCRP (mg/L) | | | | | | | | | | |
| ΔPhysicians global assessment (0-100 mm VAS) | | | | | | | | | | |
| ΔPatient global assessment (0-100 mm VAS) | | | | n.a. | | n.a. | | n.a. | | n.a. |
| Continuation of treatment, n (%) | | | | | | | | | | |
| ***Safety/Harms:*** | | | | | | | | | | |
| Withdrawals, n (%) | | | | n.a. | | n.a. | | n.a. | | n.a. |
| Withdrawals due to adverse events, n (%) | | | | n.a. | | n.a. | | n.a. | | n.a. |
| Serious adverse events (SAEs), n (%) | | | | n.a. | | n.a. | | n.a. | | n.a. |

Summary of sensitivity analyses to be conducted. Each sensitivity analysis will be adjusted similarly as primary analysis. *Key secondary outcomes are interpreted based on the Benjamini Hochberg approach. HFLM; High fiber/low meat group, LFHM; Low fiber/high meat, CI; confidence interval, HBI; Harvey Bradshaw Index, Mayo; Mayo Clinic Score, ACR20; 20% improvement according to the criteria of the American College of Rheumatology, ASAS20; 20% improvement according to Assessment of Spondyloarthritis International Society, PASI75; 75% improvement in the Psoriasis Area and Severity Index, CRP; C-reactive protein, VAS; visual analog scale, SF-12; 12-item Short Form survey, PCS; physical component summary, MCS; mental component summary.

Appendix Table 3. Secondary explorative analyses; comparison of high vs. low fiber intake.

| | Unadjusted (crude) model[1] | | | | Adjusted model[2] | | Propensity adjusted model[3] | |
|---|---|---|---|---|---|---|---|---|
| **Outcome** | **HF** | **LF** | **Difference (95% CI)** | **P value*** | **Difference (95% CI)** | **P value*** | **Difference (95% CI)** | **P value*** |
| **Primary outcome (Composite outcome)** | | | | | | | | |
| Clinical response, n (%) | | | | | | | | |
| ***Sub-components*** | | | | | | | | |
| HBI≤4, n (%) | | | | n.a. | | n.a. | | n.a. |
| Mayo≤2, n (%) | | | | n.a. | | n.a. | | n.a. |
| ACR20 response, n (%) | | | | n.a. | | n.a. | | n.a. |
| ASAS20 response, n (%) | | | | n.a. | | n.a. | | n.a. |
| PASI75 response, n (%) | | | | n.a. | | n.a. | | n.a. |
| ***Key secondary outcomes:*** | | | | | | | | |
| *Health-related quality of life:* | | | | | | | | |
| ΔSF-12 PCS (0-100) | | | | | | | | |
| ΔSF-12 MCS (0-100) | | | | | | | | |
| ΔSymptom burden (0-100) | | | | | | | | |
| ΔFunctional status (0-100) | | | | | | | | |
| ΔDisease-related burden (0-100) | | | | | | | | |
| ΔGeneral well-being (0-100) | | | | | | | | |
| ΔCRP (mg/L) | | | | | | | | |
| ΔPhysicians global assessment (0-100 mm VAS) | | | | | | | | |
| ΔPatient global assessment (0-100 mm VAS) | | | | n.a. | | n.a. | | n.a. |
| Continuation of treatment, n (%) | | | | | | | | |
| ***Safety/Harms:*** | | | | | | | | |
| Withdrawals, n (%) | | | | n.a. | | n.a. | | n.a. |
| Withdrawals due to adverse events, n (%) | | | | n.a. | | n.a. | | n.a. |
| Serious adverse events (SAEs), n (%) | | | | n.a. | | n.a. | | n.a. |

*Key secondary outcomes are interpreted by the Benjamini Hochberg approach. [1]Adjusted for CID type. [2]Adjusted for CID type, age, sex, clinical center, smoking and meat intake. [3]Adjusted for CID type, age, sex, clinical center, smoking, meat intake and propensity score. HF; High fiber (upper 33.3% of the study sample with regard to fiber intake), LF; Low fiber (lower 66.6% of the study sample with regard to fiber intake), CI; confidence interval, HBI; Harvey Bradshaw Index, Mayo; Mayo Clinic Score, ACR20; 20% improvement according to the criteria of the American College of Rheumatology, ASAS20; 20% improvement according to Assessment of Spondyloarthritis International Society, PASI75; 75% improvement in the Psoriasis Area and Severity Index, CRP; C-reactive protein, VAS; visual analog scale, SF-12; 12-item Short Form survey, PCS; physical component summary, MCS; mental component summary.

Appendix Table 4. Secondary explorative analyses; comparison of low vs. high red/processed meat intake.

| | Unadjusted (crude) model[1] | | | | Adjusted model[2] | | Propensity adjusted model[3] | |
|---|---|---|---|---|---|---|---|---|
| **Outcome** | **LM** | **HM** | **Difference (95% CI)** | **P value*** | **Difference (95% CI)** | **P value*** | **Difference (95% CI)** | **P value*** |
| **Primary outcome (Composite outcome)** | | | | | | | | |
| Clinical response, n (%) | | | | | | | | |
| ***Sub-components*** | | | | | | | | |
| HBI≤4, n (%) | | | | n.a. | | n.a. | | n.a. |
| Mayo≤2, n (%) | | | | n.a. | | n.a. | | n.a. |
| ACR20 response, n (%) | | | | n.a. | | n.a. | | n.a. |
| ASAS20 response, n (%) | | | | n.a. | | n.a. | | n.a. |
| PASI75 response, n (%) | | | | n.a. | | n.a. | | n.a. |
| ***Key secondary outcomes:*** | | | | | | | | |
| *Health-related quality of life:* | | | | | | | | |
| ΔSF-12 PCS (0-100) | | | | | | | | |
| ΔSF-12 MCS (0-100) | | | | | | | | |
| ΔSymptom burden (0-100) | | | | | | | | |
| ΔFunctional status (0-100) | | | | | | | | |
| ΔDisease-related burden (0-100) | | | | | | | | |
| ΔGeneral well-being (0-100) | | | | | | | | |
| ΔCRP (mg/L) | | | | | | | | |
| ΔPhysicians global assessment (0-100 mm VAS) | | | | | | | | |
| ΔPatient global assessment (0-100 mm VAS) | | | | n.a. | | n.a. | | n.a. |
| Continuation of treatment, n (%) | | | | | | | | |
| ***Safety/Harms:*** | | | | | | | | |
| Withdrawals, n (%) | | | | n.a. | | n.a. | | n.a. |
| Withdrawals due to adverse events, n (%) | | | | n.a. | | n.a. | | n.a. |
| Serious adverse events (SAEs), n (%) | | | | n.a. | | n.a. | | n.a. |

*Key secondary outcomes are interpreted based on the Benjamini Hochberg approach. [1]Adjusted for CID type. [2]Adjusted for CID type, age, sex, smoking, clinical center, and fiber intake. [3]Adjusted for CID type, age, sex, smoking, clinical center, fiber intake, and propensity score. LM; Low meat (lower 33.3 % of the study sample with regard to red/processed meat intake), HM; High meat (upper 66.6% of the study sample with regard to red/processed meat intake), CI; confidence interval, HBI; Harvey Bradshaw Index, Mayo; Mayo Clinic Score, ACR20; 20% improvement according to the criteria of the American College of Rheumatology, ASAS20; 20% improvement according to Assessment of Spondyloarthritis International Society, PASI75; 75% improvement in the Psoriasis Area and Severity Index, CRP; C-reactive protein, VAS; visual analog scale, SF-12; 12-item Short Form survey, PCS; physical component summary, MCS; mental component summary.
