# Supplementary material for: Impact of fibre and red/processed meat intake on treatment outcomes among patients with chronic inflammatory diseases initiating biological therapy: A prospective cohort study
Source: Front Nutr. 2022 Oct 13;9:985732. doi: 10.3389/fnut.2022.985732 (PMC9609158; doi:10.3389/fnut.2022.985732)
Supplement: Supplementary file 2 [file Data_Sheet_2.pdf]

Supplementary Material

Table S1. Other secondary (non-generic) outcomes. Values are medians (interquartile ranges) unless otherwise specified

Outcome	N(HFLM)	HFLM	N(LFHM)	LFHM
**Crohn's disease**				
ΔHBI, score	13	-1 (-3;0)	40	-2 (-4.5;0)
ΔNo. of draining fistulas	10	0	24	0
STRIDE remission, n (%)	3	0 (0.0)	10	4 (40.0)
Cortico-steroid free remission, n (%)	13	6 (46.2)	40	18 (45.0)
ΔConcomitant medication	11	-0.18 (-1;0)	31	-0.19 (-1;0)
**Ulcerative colitis**				
ΔMayo Clinic Score (0-12)	14	-7 (-8;-4)	21	-4 (-6;-2)
Mayo Clinic Response, n (%)	14	12 (85.7)	21	16 (76.2)
ΔSCCAI, score (0-19)	16	-5 (-7;-3)	22	-1.5 (-5;0)
STRIDE remission, n (%)	10	7 (70.0)	11	7 (63.6)
Cortico-steroid free remission, n (%)	14	9 (64.3)	21	13 (61.9)
ΔConcomitant medication	13	-0.69 (-1;0)	15	-1.33 (-2;-1)
**Rheumatoid Arthritis**				
ΔSDAI, score (0-86)	14	-13.34 (-18.71;-9.91)	17	-6.57 (-11.5;-4.10)
ΔSwollen joint count (0-28)	17	-2 (-4;-1)	18	-2 (-3;-1)
ΔTender joint count (0-28)	17	-3 (-5;-1)	18	-1 (-3;0)
ΔHAQ-DI, score (0-3)	17	-0.25 (-0.625;-0.125)	17	-0.25 (-0.5;0.0)
ΔDAS28-CRP, score (0-9.4)	14	-1.64 (-2.12;-0.77)	17	-0.85 (-1.71;-0.25)
**Axial Spondyloarthritis**				
ΔBASMI (0-10)	8	0 (-1;0.5)	17	-1 (-1;0)
ΔBASFI (0-100)	8	-15.8 (-29.60;-3.25)	15	-14.2 (-31.50;-3.40)
ΔBASDAI (0-100)	8	-25.15 (-47.55;-14.90)	15	-23.40 (-49.9;-9.10)
ΔTotal score for back pain (0-100 mm VAS)	8	-17 (-53.50;-3.50)	15	-26 (-60.0;-11.0)
**Psoriatic Arthritis**				
ΔSDAI, score (0-86)	6	-13.56 (-20.10;-6.81)	18	-9.72 (-15.19;-4.52)
ΔSwollen joint count (0-28)	6	-1.5 (-3;0)	18	-2 (-3;0)
ΔTender joint count (0-28)	6	-2.5 (-4;-2)	18	-2 (-4;-1)
ΔHAQ-DI, score (0-3)	6	0 (-0.125;0.0)	18	-0.31 (-0.625;0.0)
ΔDAS28-CRP, score (0-9.4)	6	-1.41 (-2.00;-0.61)	17	-1.63 (-2.48;-0.60)
ΔPASI score (0-72)	6	0 (-2.10;0.0)	18	-0.4 (-1.5;0.0)
Δ Psoriatic arthritis pain (100 mm VAS)	6	-47.50 (-57.0;-5.0)	16	-27.0 (-44.5;-10.5)
**Psoriasis**				
ΔPASI score (0-72)	1	-0.80	6	-6.10 (-8.5;-2.0)
Δ Psoriatic arthritis pain (100 mm VAS)	1	46	4	-27.00 (-62.0;0.0)
ΔDLQI (0-30)	1	-1	5	-4, 00 (-16.0;-1.0)

HFLM; high fiber low meat, LFHM; low fiber high meat, CI; confidence interval, HBI; Harvey Bradshaw Index, STRIDE; Selecting Therapeutic Targets in Inflammatory Bowel Disease, SCCAI; Simple Clinical Colitis Activity Index, SDAI; Simplified Disease Activity Index, HAQ-DI; Health Assessment Questionnaire Disability Index, DAS28-CRP; Disease Activity Score 28 - C-Reactive Protein, BASMI; Bath Ankylosing Spondylitis Metrology Index, BASFI; Bath Ankylosing Spondylitis Functional Index, BASDAI; Bath Ankylosing Spondylitis Disease Activity Index, PASI; Psoriasis Area and Activity index, DLQI; Dermatology Life Quality Index.

Table S2. Sensitivity analysis, complete case. Values are numbers (percentages) with odds ratios (95% CI) for dichotomous outcomes and least squares mean with differences (95% CI) for the continuous variables.

					Crude model[1]		Adjusted model[2]	
**Outcome**	**N (HFLM)**	**HFLM**	**N (LFHM)**	**LFHM**	**Contrast (95% CI)**	**P value***	**Contrast (95% CI)**	**P value***
**Primary outcome (Composite outcome)**								
Clinical response	59	39 (66.10)	117	69 (58.97)	1.39 (0.71;2.72)	0.34	1.53 (0.73;3.18)	0.260
Sub-components								
Crohns Disease, HBI≤4	13	8 (61.54)	40	22 (55.00)	1.31 (0.36;4.70)	n.a.	1.10 (0.24;4.94)	n.a.
Ulcerative Colitis, Mayo≤2	14	10 (71.43)	21	13 (61.90)	1.54 (0.36;6.60)	n.a.	2.97 (0.52;17.05)	n.a.
Rheumatoid Arthritis, ACR20 response	17	14 (82.35)	17	6 (35.29)	8.56 (1.74;42.17)	n.a.	8.97 (1.19;67.40)	n.a.
Axial Spondyloarthritis, ASAS20 response	8	4 (50.00)	15	10 (66.67)	0.5 (0.09;2.89)	n.a.	0.66 (0.03;15.00)	n.a.
Psoriatic Arthritis, ACR20 response	6	3 (50.00)	18	13 (72.22)	0.38 (0.06;2.58)	n.a.	0.19 (0.02;2.23)	n.a.
Psoriasis, PASI75 response	1	0 (0.00)	6	5 (83.33)	n.a.	n.a.	n.a.	n.a.
Key secondary outcomes:								
ΔSF-12 PCS (0-100)	56	-1.78	98	-1.15	-0.63 (-2.03;0.78)	0.38	-0.54 (-2.06;0.98)	0.49
ΔSF-12 MCS (0-100)	56	2.25	98	0.89	1.36 (-0.44;3.16)	0.14	1.19 (-0.72;3.10)	0.220
ΔCRP (mg/L)	43	-0.73	89	-1.83	1.11 (-9.56;11.78)	0.84	0.69 (-10.88;12.25)	0.907
ΔPhysicians global assessment (0-100 mm VAS)	39	-34.63	67	-34.71	0.08 (-8.28;8.44)	0.99	-0.24 (-9.26;8.77)	0.957

Complete case sensitivity analysis (logistic regression). Complete case = no missing data on the specific outcome (no imputation of data). [1]The crude model is only adjusted for CID and for the continouos outcomes also the baseline value of the outcome of interest. [2]The adjusted model is adjusted for CID, age, sex, smoking status and for the continous outcomes also the baseline value.

*Key secondary outcomes are interpreted based on the Hochberg sequential procedure. HFLM; High fiber/low meat group, LFHM; Low fiber/high meat, CI; confidence interval, HBI; Harvey Bradshaw Index, Mayo; Mayo Clinic Score, ACR20; 20% improvement according to the criteria of the American College of Rheumatology, ASAS20; 20% improvement according to Assessment of Spondyloarthritis International Society, PASI75; 75% improvement in the Psoriasis Area and Severity Index, CRP; C-reactive protein, VAS; visual analog scale, SF-12; 12-item Short Form survey, PCS; physical component summary, MCS; mental component summary.

Table S3. Sensitivity analysis, per protocol analysis. Values are numbers (percentages) and odds ratios for dichotomous outcomes and least squares mean differences for the continuous variables.

					Crude model[1]		Adjusted model[2]	
**Outcome**	**N (HFLM)**	**HFLM**	**N (LFHM)**	**LFHM**	**Difference (95% CI)**	**P value***	**Difference (95% CI)**	**P value***
**Primary outcome (Composite outcome)**								
Clinical response, n (%)	59	39 (66.10)	109	68 (61.82)	1.18 (0.60;2.35)	0.632	1.26 (0.60;2.68)	0.543
Sub-components								
Crohns Disease, HBI≤4	13	8 (61.54)	38	22 (56.41)	1.16 (0.32;4.23)	n.a.	1.01 (0.22;4.61)	n.a.
Ulcerative Colitis, Mayo≤2	14	10 (71.43)	20	12 (60.00)	1.67 (0.39;7.21)	n.a.	3.07 (0.53;17.68)	n.a.
Rheumatoid Arthritis, ACR20 response	17	14 (82.35)	14	6 (42.86)	6.22 (1.21;31.94)	n.a.	5.97 (0.69;51.82)	n.a.
Axial Spondyloarthritis, ASAS20 response	4	4 (50.00)	10	10 (66.67)	0.50 (0.09;2.89)	n.a.	0.66 (0.03;15.00)	n.a.
Psoriatic Arthritis, ACR20 response	6	3 (50.00)	16	13 (81.25)	0.23 (0.03;1.76)	n.a.	n.a.	n.a.
Psoriasis, PASI75 response	1	0 (0.00)	6	5 (83.33)	n.a.	n.a.	n.a.	n.a.
Key secondary outcomes:								
ΔSF-12 PCS (0-100)	56	-1.70	92	-1.15	-0.56 (-2.00;0.89)	0.448	-0.44 (-2.00;1.12)	0.579
ΔSF-12 MCS (0-100)	56	2.23	92	0.95	1.27 (-0.54;3.08)	0.168	1.05 (-0.87;2.97)	0.282
ΔCRP (mg/L)	42	-1.20	83.00	-3.22	2.02 (-8.46;12.50)	0.703	1.35 (-9.95;12.65)	0.813
ΔPhysicians global assessment (0-100 mm VAS)	39	-35.54	61	-35.43	-0.11 (-8.52;8.30)	0.980	-0.87 (-9.94;8.20)	0.850

Sensitivity analysis on the per protocol population (logistic regression). In the per protocol population the following participants were excluded; patients that were not treated with biologics, patients treated for less than 70 days, RA and PsA patients with zero swollen or tender joints at baseline or patients lacking the outcome of interest. [1]The crude model is only adjusted for CID and for the continouos outcomes also the baseline value of the outcome of interest. [2]The adjusted model is adjusted for CID, age, sex, smoking status and for the continous outcomes also the baseline value.
*Key secondary outcomes are interpreted based on the Hochberg sequential procedure. HFLM; High fiber/low meat group, LFHM; Low fiber/high meat, CI; confidence interval, HBI; Harvey Bradshaw Index, Mayo; Mayo Clinic Score, ACR20; 20% improvement according to the criteria of the American College of Rheumatology, ASAS20; 20% improvement according to Assessment of Spondyloarthritis International Society, PASI75; 75% improvement in the Psoriasis Area and Severity Index, CRP; C-reactive protein, VAS; visual analog scale, SF-12; 12-item Short Form survey, PCS; physical component summary, MCS; mental component summary.

Table S4. Sensitivity analysis, non-responder imputation. Values are numbers (percentages) and odds ratios for dichotomous outcomes and least squares mean differences for the continuous variables.

			Crude model[1]		Adjusted model[2]	
**Outcome**	**HFLM (N=64)**	**LFHM (N=129)**	**Difference (95% CI)**	**P value***	**Difference (95% CI)**	**P value***
**Primary outcome (Composite outcome)**						
Clinical response, n (%)	39 (60.94)	69 (53.49)	1.39 (0.75;2.61)	0.298	1.41 (0.71;2.78)	0.323
Sub-components						
Crohns Disease, HBI≤4	8 (53.33)	22 (55.00)	0.94 (0.28;3.07)	n.a.	0.68 (0.18;2.65)	n.a.
Ulcerative Colitis, Mayo≤2	10 (58.82)	13 (56.52)	1.10 (0.31;3.91)	n.a.	1.34 (0.31;5.94)	n.a.
Rheumatoid Arthritis, ACR20 response	14 (82.35)	6 (30.00)	10.89 (2.26;52.42)	n.a.	11.29 (1.59;80.25)	n.a.
Axial Spondyloarthritis, ASAS20 response	4 (50.00)	10 (50.00)	1.00 (0.19;5.15)	n.a.	0.64 (0.03;16.01)	n.a.
Psoriatic Arthritis, ACR20 response	3 (50.00)	13 (72.22)	0.38 (0.06;2.58)	n.a.	0.19 (0.02;2.23)	n.a.
Psoriasis, PASI75 response	0 (0.00)	5 (62.50)	n.a.	n.a.	n.a.	n.a.
Key secondary outcomes:						
ΔSF-12 PCS (0-100)	-1.49	-0.91	-0.58 (-1.86;0.71)	0.378	-0.66 (-2.02;0.71)	0.346
ΔSF-12 MCS (0-100)	1.64	0.84	0.80 (-0.87;2.47)	0.345	0.77 (-1.01;2.54)	0.394
ΔCRP (mg/L)	-1.34	-1.1	-0.23 (-8.76;8.29)	0.957	-0.77 (-9.93;8.39)	0.868
ΔPhysicians global assessment (0-100 mm VAS)	-30.7	-29.45	-1.23 (-9.97;7.50)	0.780	-0.32 (-9.80;9.15)	0.946

Non-responder imputation sensitivity analysis (logistic regression). Missing outcome data were imputed as non-response. [1]The crude model is only adjusted for CID and for the continuous outcomes also the baseline value of the outcome of interest. [2]The adjusted model is adjusted for CID, age, sex, smoking status and for the continuous outcomes also the baseline value.
*Key secondary outcomes are interpreted based on the Hochberg sequential procedure. HFLM; High fibre/low meat group, LFHM; Low fibre/high meat, CI; confidence interval, HBI; Harvey Bradshaw Index, Mayo; Mayo Clinic Score, ACR20; 20% improvement according to the criteria of the American College of Rheumatology, ASAS20; 20% improvement according to Assessment of Spondyloarthritis International Society, PASI75; 75% improvement in the Psoriasis Area and Severity Index, CRP; C-reactive protein, VAS; visual analog scale, SF-12; 12-item Short Form survey, PCS; physical component summary, MCS; mental component summary.

Table S5. Sensitivity analysis on the population fitting the unmodified original eligibility criteria. Values are numbers (percentages) and odds ratios for dichotomous outcomes and least squares mean differences for the continuous variables.

					Crude model[1]		Adjusted model[2]	
**Outcome**	**N (HFLM)**	**HFLM**	**N (LFHM)**	**LFHM**	**Difference (95% CI)**	**P value***	**Difference (95% CI)**	**P value***
**Primary outcome (Composite outcome)**								
Clinical response, n (%)	51	37 (71.76)	98	56 (57.35)	2.13 (0.97;4.68)	0.059	2.61 (1.09;6.18)	0.032
Sub-components								
Crohns Disease, HBI≤4	9	6 (66.67)	25	16 (64.00)	1.12 (0.23;5.62)	n.a.	1.00 (0.15;6.60)	n.a.
Ulcerative Colitis, Mayo≤2	12	10 (80)	17	9 (52.94)	3.70 (0.51;26.73)	n.a.	8.78 (0.63;121.59)	n.a.
Rheumatoid Arthritis, ACR20 response	17	14 (82.35)	18	6 (33.33)	9.33 (1.91;45.58)	n.a.	10.14 (1.42;72.51)	n.a.
Axial Spondyloarthritis, ASAS20 response	8	4 (50.00)	20	11 (56.00)	0.78 (0.14;4.51)	n.a.	0.59 (0.02;14.56)	n.a.
Psoriatic Arthritis, ACR20 response	5	3 (60.00)	17	13 (76.47)	0.46 (0.06;3.81)	n.a.	0.33 (0.02;4.86)	n.a.
Psoriasis, PASI75 response	0	n.a.	1	1 (100.00)	n.a.	n.a.	n.a.	n.a.
Key secondary outcomes:								
ΔSF-12 PCS (0-100)	51	-1.75	98	-1.39	-0.36 (-1.68;0.97)	0.598	-0.49 (-1.87;0.89)	0.488
ΔSF-12 MCS (0-100)	51	2.17	98	0.63	1.54 (-0.12;3.20)	0.068	1.60 (-0.15;3.34)	0.073
ΔCRP (mg/L)	51	-1.02	98	-3.4	2.38 (-5.53;10.30)	0.555	1.57 (-6.74;9.87)	0.711
ΔPhysicians global assessment (0-100 mm VAS)	51	-43.41	98	-43.79	0.38 (-6.65;7.41)	0.915	1.16 (-6.08;8.41)	0.753

Sensitivity analysis (logistic regression) on the population fitting the original unmodified criteria i.e. patients naïve to biological treatment and treated with TNF inhibitor. [1]The crude model is only adjusted for CID and for the continouos outcomes also the baseline value of the outcome of interest. [2]The adjusted model is adjusted for CID, age, sex, smoking status and for the continous outcomes also the baseline value.
*Key secondary outcomes are interpreted based on the Hochberg sequential procedure. HFLM; High fiber/low meat group, LFHM; Low fiber/high meat, CI; confidence interval, HBI; Harvey Bradshaw Index, Mayo; Mayo Clinic Score, ACR20; 20% improvement according to the criteria of the American College of Rheumatology, ASAS20; 20% improvement according to Assessment of Spondyloarthritis International Society, PASI75; 75% improvement in the Psoriasis Area and Severity Index, CRP; C-reactive protein, VAS; visual analog scale, SF-12; 12-item Short Form survey, PCS; physical component summary, MCS; mental component summary.

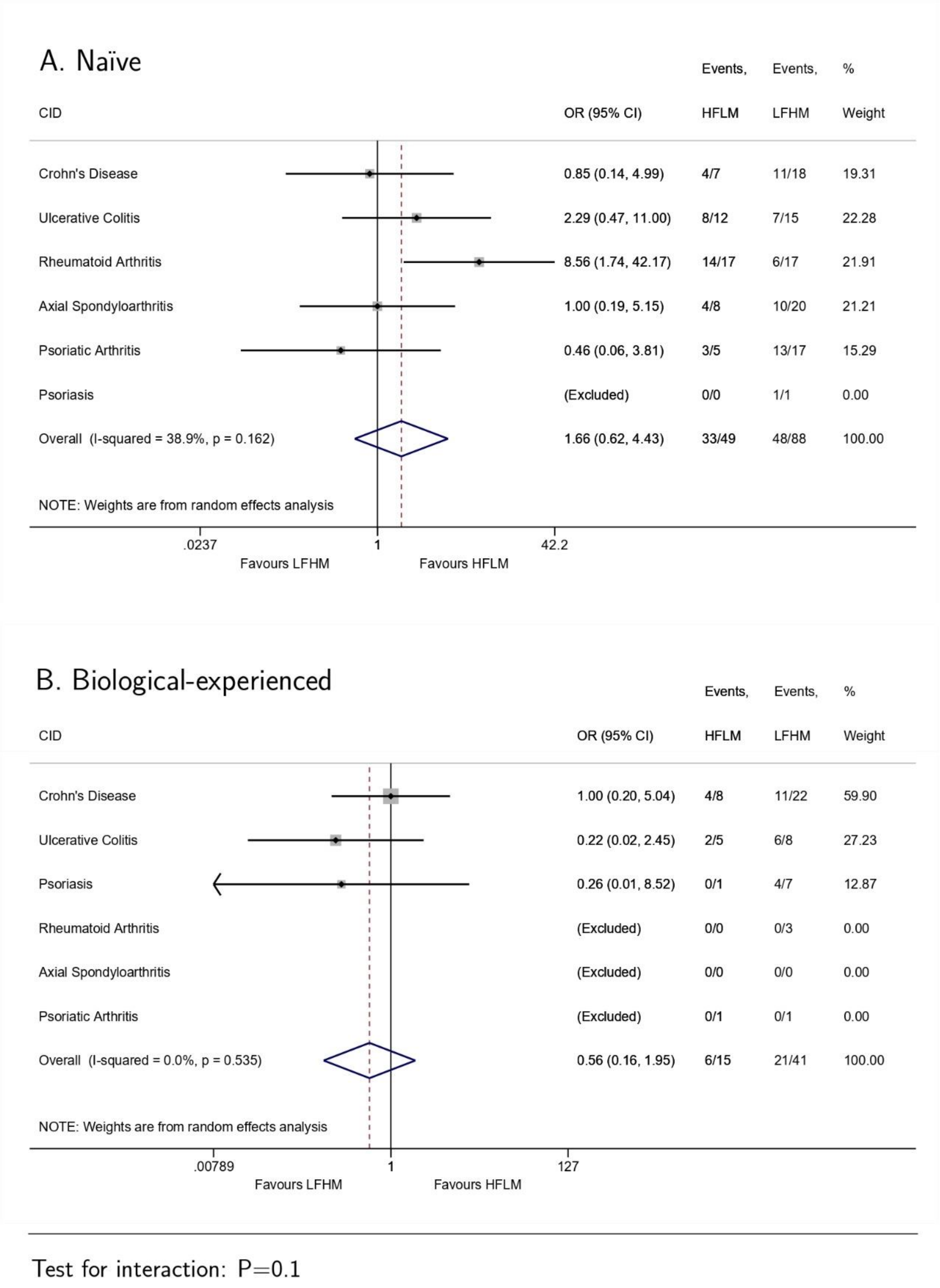

Fig. S1. Meta-analysis (randoms effects) of the biological naïve subgroup and the biological-experienced subgroup comparing clinical response to biologics between the HFLM and LFHM groups.

Non-respnder imputation is applied for missing values. The horizontal lines represent the odds ratio (OR) ± 95% confidence interval. Event= clinical response according to the specified criteria for each CID, i.e. the number shows how many out of the total number of participants in the group, that have had a clinical response. Test for interaction is a subgroup analysis comparing the two subgroups [34, 35].
HFLM: High fibre and low meat, LFHM: low fibre and high meat.

Table S6. Explorative analysis. High fibre intake (HF) versus low fibre intake (LF). Values are numbers (percentages) and odds ratios for dichotomous outcomes and least squares mean differences for the continuous variables.

					Crude model[1]		Adjusted model[2]	
**Outcome**	**N (HF)**	**HF**	**N (LF)**	**LF (N=129)**	**Difference (95% CI)**	**P value***	**Difference (95% CI)**	**P value***
**Primary outcome (Composite outcome)**								
Clinical response, n (%)	64	40 (62.81)	129	73 (56.90)	1.30 (0.66;2.57)	0.446	1.19 (0.57;2.47)	0.639
Sub-components								
Crohns Disease, HBI≤4	11	8 (72.73)	44	22 (50.00)	2.67 (0.62;11.40)	n.a.	2.10 (0.34;12.85)	n.a.
Ulcerative Colitis, Mayo≤2	17	13 (75.29)	23	13 (56.52)	2.15 (0.49;9.46)	n.a.	2.26 (0.44;11.66)	n.a.
Rheumatoid Arthritis, ACR20 response	14	9 (64.29)	23	12 (50.43)	1.77 (0.44;7.04)	n.a.	1.43 (0.28;7.39)	n.a.
Axial Spondyloarthritis, ASAS20 response	14	7 (47.14)	14	9 (61.43)	0.56 (0.10;3.13)	n.a.	0.28 (0.02;5.23)	n.a.
Psoriatic Arthritis, ACR20 response	6	4 (66.67)	18	12 (66.67)	1 (0.14;7.10)	n.a.	0.52 (0.04;6.48)	n.a.
Psoriasis, PASI75 response	2	0 (0.00)	7	6 (85.71)	n.a.	n.a.	n.a.	n.a.
Key secondary outcomes:								
Health-related quality of life:								
ΔSF-12 PCS (0-100)	64	-1.5	129	-1.34	-0.16 (-1.33;1.01)	0.79	-0.27 (-1.47;0.93)	0.658
ΔSF-12 MCS (0-100)	64	0.79	129	0.99	-0.20 (-1.74;1.34)	0.801	-0.22 (-1.78;1.34)	0.781
ΔSymptom burden (0-100)	64	-20.99	129	-25.96	4.97 (-2.08;12.02)	0.167	2.29 (-4.49;9.07)	0.508
ΔFunctional status (0-100)	64	-17.64	129	-23.00	5.36 (-2.09;12.82)	0.158	2.75 (-4.46;9.96)	0.455
ΔDisease-related burden (0-100)	64	-19.26	129	-23.02	3.76 (-3.92;11.44)	0.337	3.04 (-4.57;10.64)	0.434
ΔGeneral well-being (0-100)	64	-16.28	129	-18.42	2.14 (-4.66;8.95)	0.537	1.35 (-5.39;8.09)	0.694
ΔCRP (mg/L)	64	3.34	129	-3.99	7.33 (-0.44;15.12)	0.065	4.62 (-2.92;12.16)	0.23
ΔPhysicians global assessment (0-100 mm VAS)	64	-41.63	129	-41.87	0.25 (-7.03;7.53)	0.947	0.17 (-7.10;7.44)	0.964
Continuation of treatment, n (%)	64	55 (85.94)	129	106 (82.48)	1.37 (0.58;3.28)	0.473	1.34 (0.53;3.39)	0.533

Explorative analysis; High fibre intake (HF= the upper tertile of the study sample) versus low fibre intake (LF=the two lower tertiles of the study sample with regard to fibre intake). [1]Adjusted only for CID in the dichotomous outcomes. For the continuous outcomes the model is also adjusted for the baseline value of the outcome of interest. [2]Adjusted for CID, age, sex, smoking-status, the intake of red/processed meat and for the continouus outcomes also the baseline value. *Key secondary outcomes are interpreted based on the Hochberg sequential procedure. HF; High fiber (the upper tertile of the study sample), LF; Low fiber (the two lower tertiles of the study sample), CI; confidence interval, HBI; Harvey Bradshaw Index, Mayo; Mayo Clinic Score, ACR20; 20% improvement according to the criteria of the American College of Rheumatology, ASAS20; 20% improvement according to Assessment of Spondyloarthritis International Society, PASI75; 75% improvement in the Psoriasis Area and Severity Index, CRP; C-reactive protein, VAS; visual analog scale, SF-12; 12-item Short Form survey, PCS; physical component summary, MCS; mental component summary.

Table S7. Explorative analysis; Low red/processed meat intake versus high red/processed meat intake. Values are numbers (percentages) and odds ratios for dichotomous outcomes and least squares mean differences for the continuous variables.

					Crude model[1]		Adjusted model[2]	
**Outcome**	**N (LM)**	**LM**	**N (HM)**	**HM**	**Difference (95% CI)**	**P value***	**Difference (95% CI)**	**P value***
**Primary outcome (Composite outcome)**								
Clinical response, n (%)	65	39 (60.31)	128	74.4 (58.13)	1.16 (0.60;2.26)	0.661	1.38 (0.67;2.87)	0.379
Sub-components								
Crohns Disease, HBI≤4	21	11 (52.38)	34	19 (55.88)	0.87 (0.29;2.59)	n.a.	0.83 (0.24; 2.87)	n.a.
Ulcerative Colitis, Mayo≤2	14	10 (70.00)	26	16 (61.54)	1.49 (0.29;7.65)	n.a.	2.06 (0.31;13.46)	n.a.
Rheumatoid Arthritis, ACR20 response	16	11 (70.00)	21	9 (44.76)	2.89 (0.68;12.30)	n.a.	3.97 (0.48;33.10)	n.a.
Axial Spondyloarthritis, ASAS20 response	8	3 (40.00)	20	12 (60.00)	0.44 (0.08;2.52)	n.a.	0.50 (0.03;8.48)	n.a.
Psoriatic Arthritis, ACR20 response	6	4 (66.67)	18	12 (66.67)	1.00 (0.14;7.10)	n.a.	1.66 (0.16;17.31)	n.a.
Psoriasis, PASI75 response	0	n.a.	9	6 (66.67)	n.a.	n.a.	n.a.	n.a.
Key secondary outcomes:								
Health-related quality of life:								
ΔSF-12 PCS (0-100)	65	-1.46	128	-1.34	-0.10 (-1.26;1.06)	0.862	-0.11 (-1.29;0.60)	0.858
ΔSF-12 MCS (0-100)	65	1.22	128	0.77	0.45 (-1.07;1.97)	0.560	0.27 (-1.27;1.82)	0.728
ΔSymptom burden (0-100)	65	-26.04	128	-23.43	-2.61 (-9.61;4.39)	0.464	-3.11 (-10.01;3.80)	0.378
ΔFunctional status (0-100)	65	-22.43	128	-20.61	-1.82 (-9.22;5.58)	0.63	-2.04 (-9.27;5.19)	0.58
ΔDisease-related burden (0-100)	65	-20.07	128	-22.64	2.57 (-4.97;10.12)	0.505	2.22 (-5.37;9.81)	0.567
ΔGeneral well-being (0-100)	65	-16.23	128	-18.46	2.22 (-4.50;8.95)	0.517	0.69 (-6.04;7.43)	0.84
ΔCRP (mg/L)	65	-0.78	128	-1.96	1.18 (-6.56;8.92)	0.765	2.23 (-5.53;10.00)	0.573
ΔPhysicians global assessment (0-100 mm VAS)	65	-36.64	128	-44.41	7.76 (0.66;14.87)	0.032	8.86 (1.74;15.98)	0.015
Continuation of treatment, n (%)	65	52 (79.69)	128	110 (85.63)	0.62 (0.27;1.42)	0.260	0.71 (0.29;1.70)	0.440

Explorative analysis; Low red/processed meat intake (LM) versus high red/processed meat intake (HM). [1]Adjusted only for CID in the dichotomous outcomes. For the continuous outcomes the model is also adjusted for the baseline value of the outcome of interest. [2]Adjusted for CID, age, sex, smoking-status, the intake of fiber and for the continouus outcomes also the baseline value. *Key secondary outcomes are interpreted based on the Hochberg sequential procedure. LM; the lower tertile of the study sample with regard to red/processed meat intake, HM; the two upper tertiles of the study sample with regard to red/processed meat intake, CI; confidence interval, HBI; Harvey Bradshaw Index, Mayo; Mayo Clinic Score, ACR20; 20% improvement according to the criteria of the American College of Rheumatology, ASAS20; 20% improvement according to Assessment of Spondyloarthritis International Society, PASI75; 75% improvement in the Psoriasis Area and Severity Index, CRP; C-reactive protein, VAS; visual analog scale, SF-12; 12-item Short Form survey, PCS; physical component summary, MCS; mental component summary.

Table S8. Additional sensitivity analysis including BMI as a covariate. Values are numbers (percentages) and odds ratios.

			Adjusted model	
**Outcome**	**HFLM (N=64)**	**LFHM (N=129)**	**Difference (95% CI)**	**P value**
**Primary outcome (Composite outcome)**				
Clinical response, n (%)	41 (64)	73 (56)	1.46 (0.71;3.03)	0.302
Sub-components				
Crohns Disease, HBI≤4	8 (53)	22 (55)	0.99 (0.15;6.58)	n.a.
Ulcerative Colitis, Mayo≤2	12 (69)	14 (61)	9.42 (0.62;143.96)	n.a.
Rheumatoid Arthritis, ACR20 response	14 (82)	7 (28)	9.83 (1.40;68.84)	n.a.
Axial Spondyloarthritis, ASAS20 response	4 (50)	11 (56)	0.72 (0.02;22.59)	n.a.
Psoriatic Arthritis, ACR20 response	3 (50)	13 (72)	0.28 (0.02;4.93)	n.a.
Psoriasis, PASI75 response	0 (0)	6 (75)	n.a.	n.a.

Sensitivity analysis including BMI as a covariate in the adjusted analysis, i.e. the analysis is adjusted for CID, age, sex, smoking status, BMI and for the continous outcomes also the baseline value.
The crude model is not shown as it is identical to the one in table 2. HFLM; High fiber/low meat group, LFHM; Low fiber/high meat, CI; confidence interval, HBI; Harvey Bradshaw Index, Mayo; Mayo Clinic Score, ACR20; 20% improvement according to the criteria of the American College of Rheumatology, ASAS20; 20% improvement according to Assessment of Spondyloarthritis International Society, PASI75; 75% improvement in the Psoriasis Area and Severity Index.

Table S9. Baseline nutrient characteristics across the six diagnoses of chronic inflammatory disease.

Macronutrient/food item	**Crohn's Disease**		**Ulcerative Colitis**		**Rheumatoid Arthritis**	
	HFLM N=15	LFHM N=40	HFLM N=17	LFHM N=23	HFLM N=17	LFHM N=20
Energy (MJ/day), mean (SD)	6733.1 (2184.6)	7624.9 (2641.5)	9035.3 (3627.9)	10173.9 (5950.3)	7041.0 (1931.7)	8097.1 (2146.6)
Fibre (g/day), mean (SD)	17.4 (5.9)	14.1 (5.9)	24.8 (11.7)	19.6 (10.6)	19.6 (4.8)	17.2 (5.5)
Red/processed meat (g/day), mean (SD)	43.2 (12.7)	92.0 (39.8)	52.9 (24.3)	154.2 (186.2)	50.1 (21.9)	110.6 (48.0)
	**Axial Spondyloarthropathy**		**Psoriatic Arthritis**		**Psoriasis**	
	HFLM N=8	LFHM N=20	HFLM N=6	LFHM N=18	HFLM N=1	LFHM N=8
Energy (MJ/day), mean (SD)	7775.3 (2727.1)	10653.7 (3639.6)	9487.1 (2927.2)	7555.5 (2223.8)	n.a.	7793.1 (2555.7)
Fibre (g/day), mean (SD)	24.0 (7.2)	19.2 (7.4)	24.9 (11.1)	14.6 (5.5)	n.a.	12.3 (5.2)
Red/processed meat (g/day), mean (SD)	50.1 (34.6)	155.3 (81.7)	64.8 (16.8)	98.1 (41.3)	n.a.	106.7 (53.0)
